# Supplementary material for: Iron-sulfur cluster loss in mitochondrial CISD1 mediates PINK1 loss-of-function phenotypes
Source: eLife. 2024 Aug 19;13:e97027. doi: 10.7554/eLife.97027 (PMC11383524; doi:10.7554/eLife.97027)
Supplement: Figure 1—source data 1. [file elife-97027-fig1-data1.zip › Figure 1 - Source data 1/Figure 1 - 1B labeled.pptx]

## Slide 1
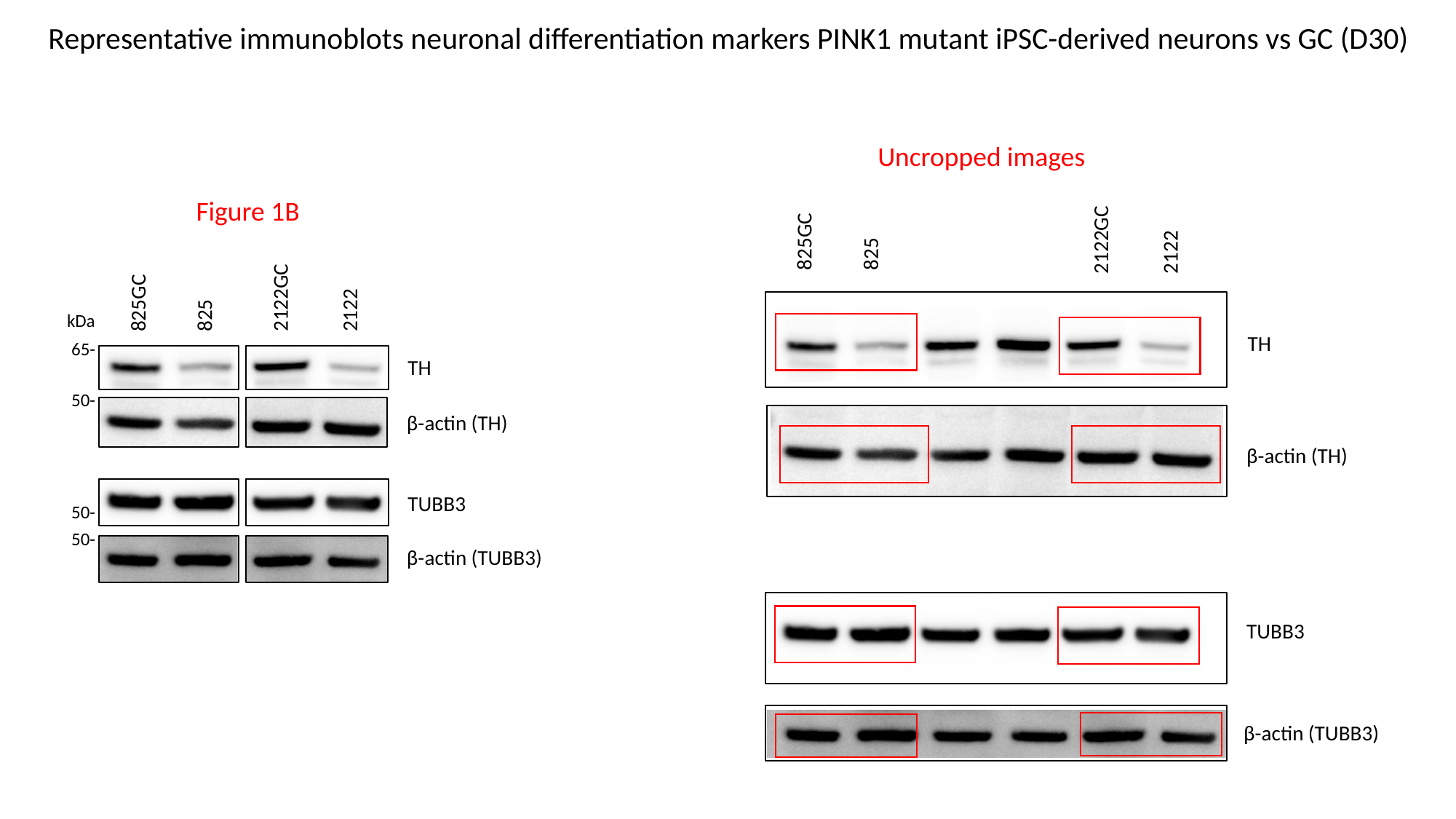

Representative immunoblots neuronal differentiation markers PINK1 mutant iPSC-derived neurons vs GC (D30)
Uncropped images
Figure 1B
2122GC
825GC
825
2122
2122GC
825GC
825
2122
TH
β-actin (TH)
TUBB3
β-actin (TUBB3)
kDa
65-
50-
50-
50-
TH
β-actin (TH)
TUBB3
β-actin (TUBB3)
