# Supplementary figures and images for: Iron-sulfur cluster loss in mitochondrial CISD1 mediates PINK1 loss-of-function phenotypes
Source: eLife. 2024 Aug 19;13:e97027. doi: 10.7554/eLife.97027 (PMC11383524; doi:10.7554/eLife.97027)

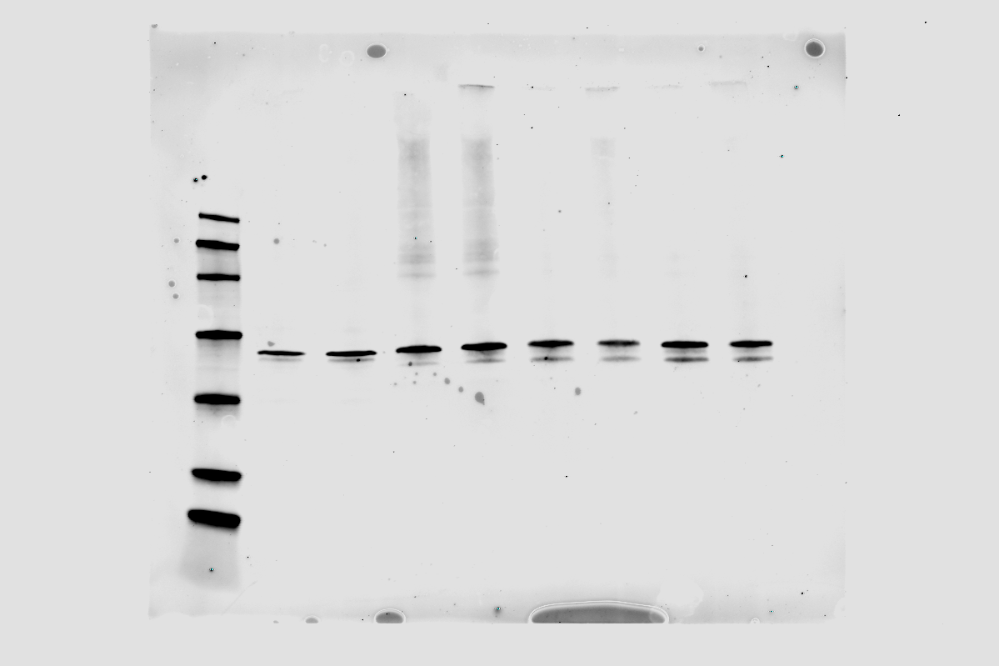

Supplement: Figure 1—source data 1. [file elife-97027-fig1-data1.zip › Figure 1 - Source data 1/Figure 1 - 1C-raw.tif]

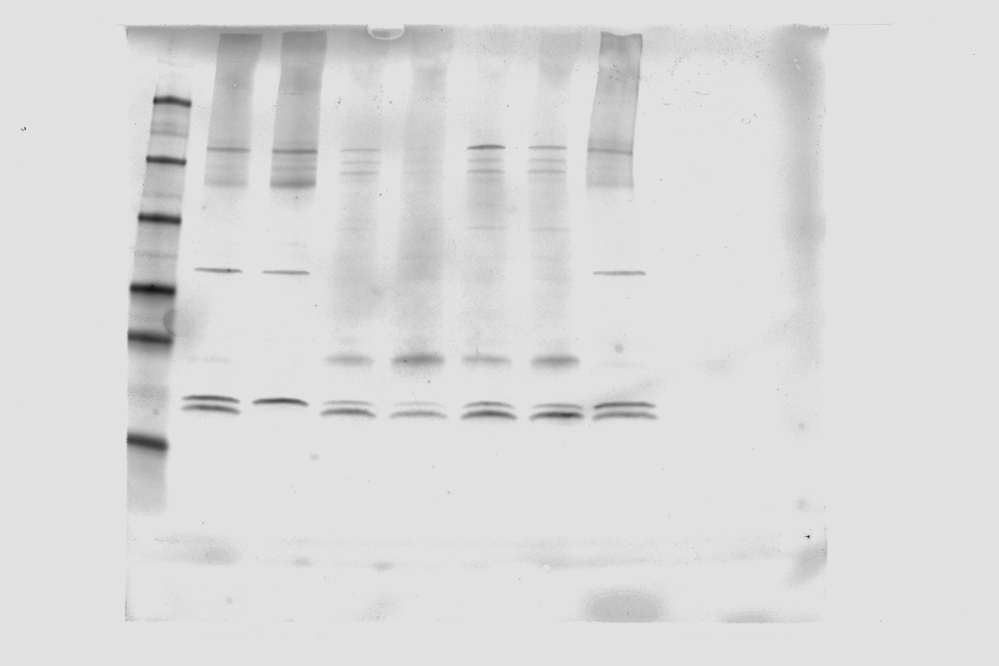

Supplement: Figure 1—source data 1. [file elife-97027-fig1-data1.zip › Figure 1 - Source data 1/Figure 1 - 1C┬┤ -raw 2.tif]

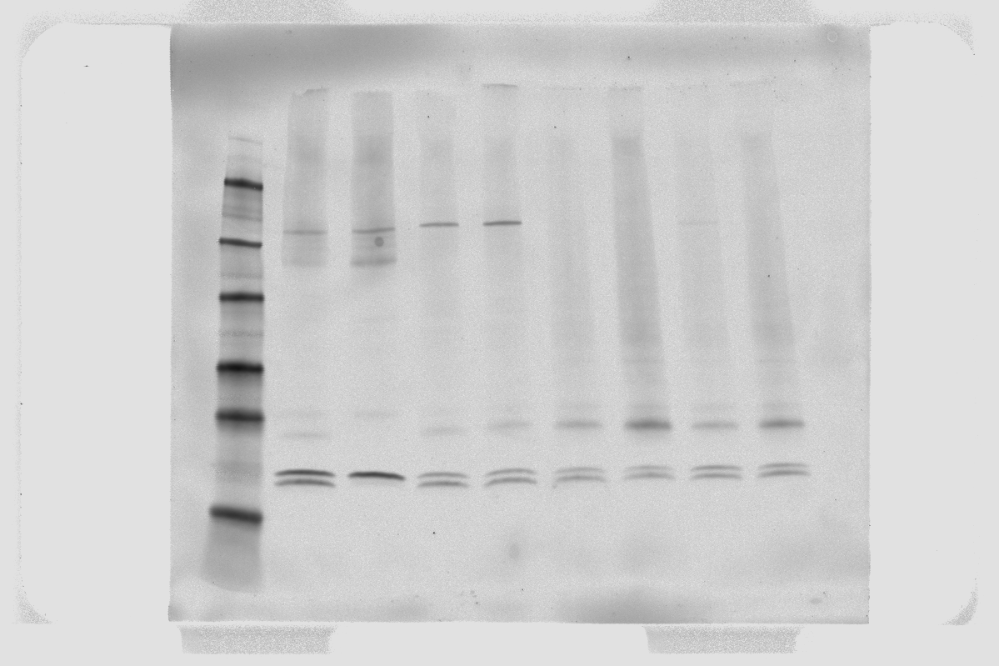

Supplement: Figure 1—source data 1. [file elife-97027-fig1-data1.zip › Figure 1 - Source data 1/Figure 1 - 1C -raw 2.tif]

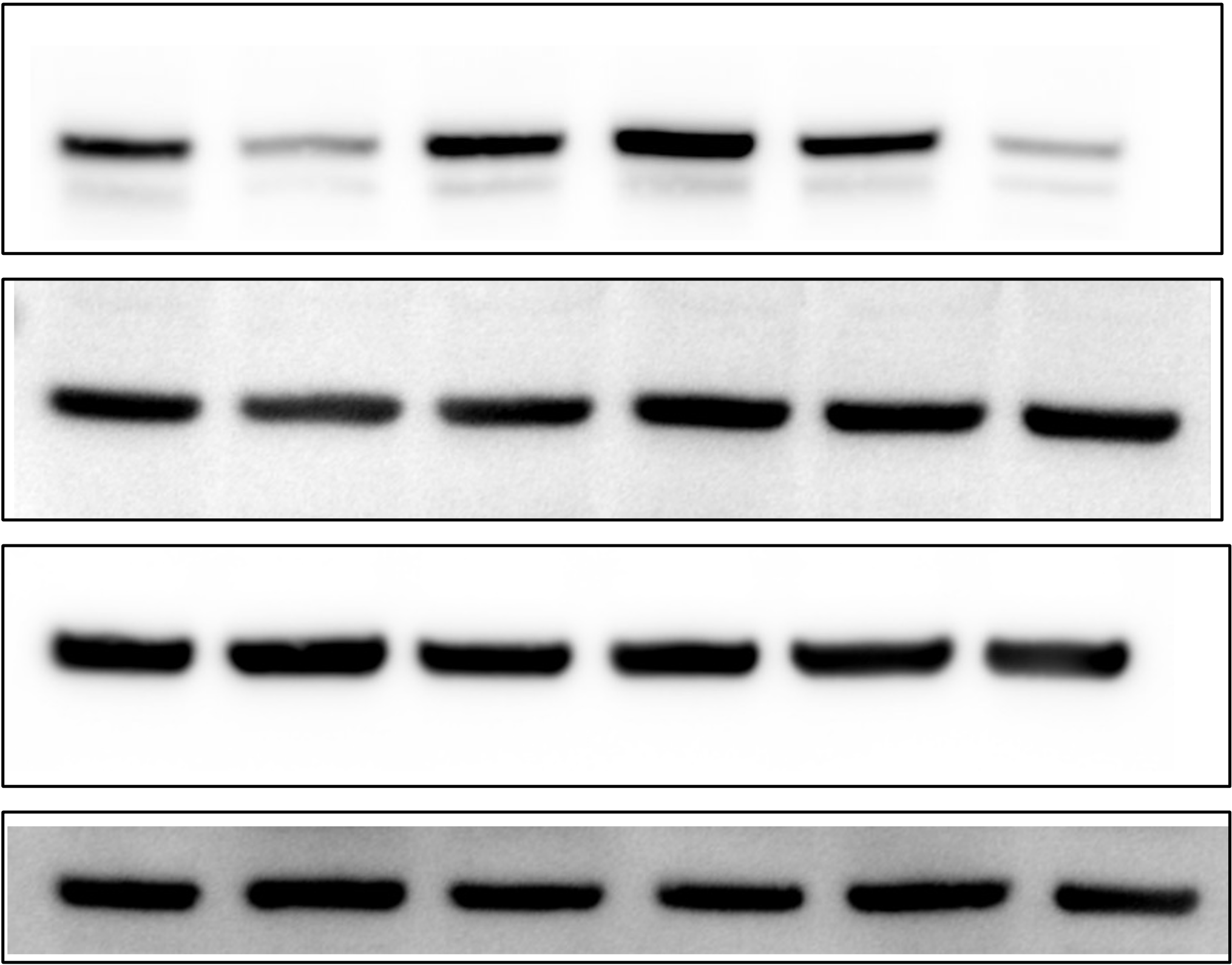

Supplement: Figure 1—source data 1. [file elife-97027-fig1-data1.zip › Figure 1 - Source data 1/Figure 1 - 1B raw.png]

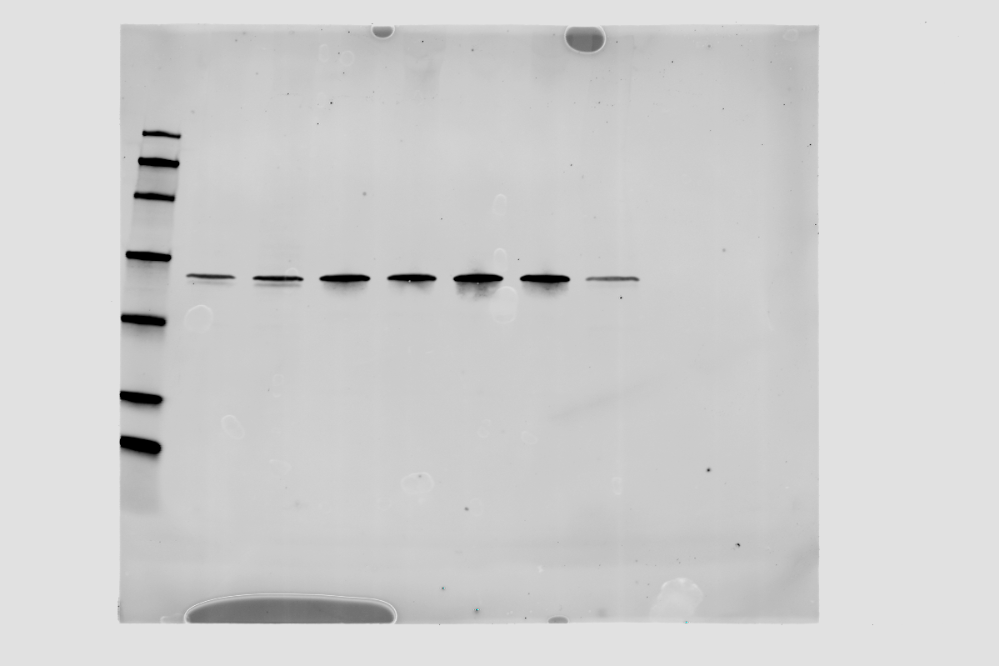

Supplement: Figure 1—source data 1. [file elife-97027-fig1-data1.zip › Figure 1 - Source data 1/Figure 1 - 1C┬┤-raw .tif]

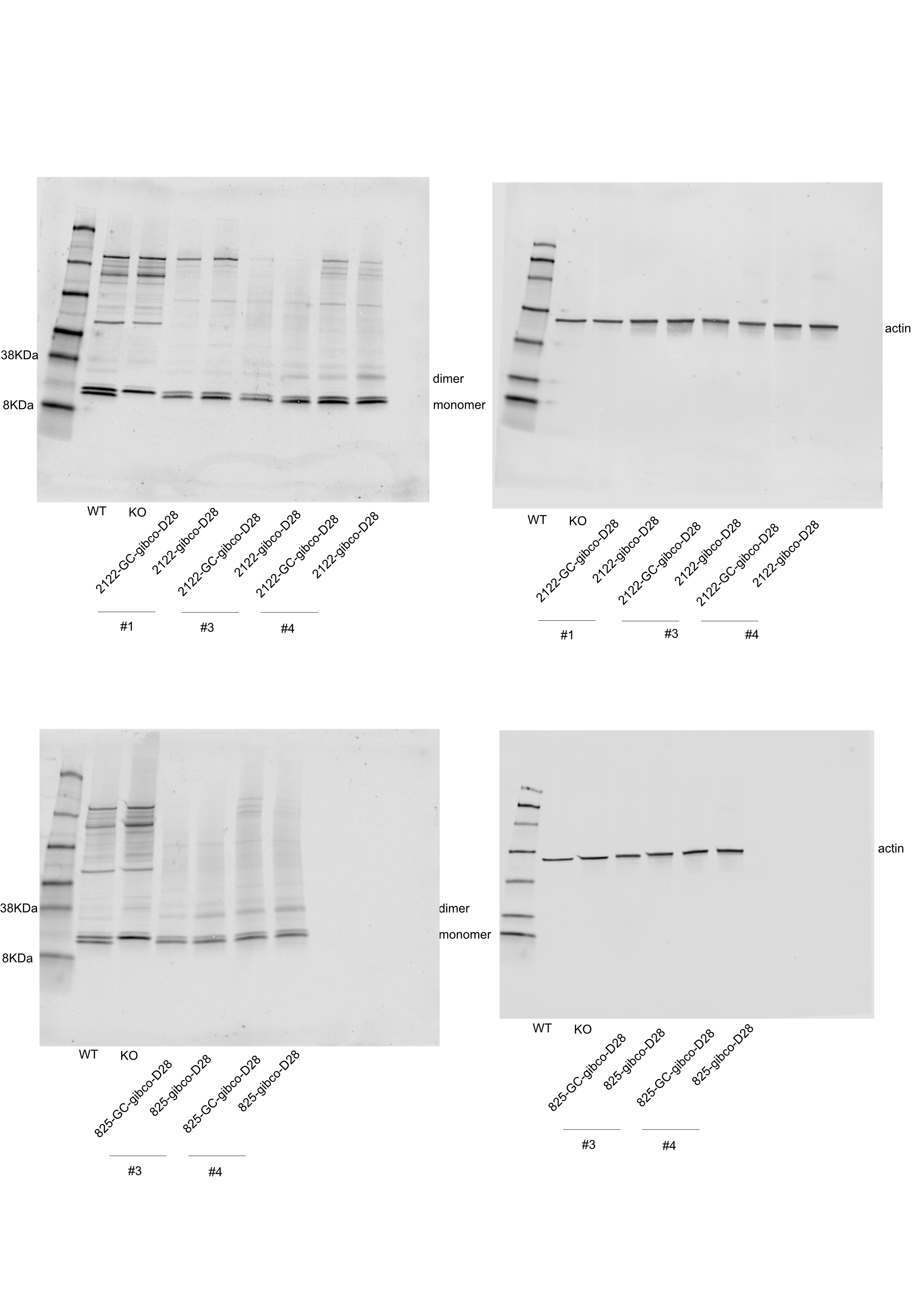

Supplement: Figure 1—source data 1. [file elife-97027-fig1-data1.zip › Figure 1 - Source data 1/Figure 1 - 1C┬┤D┬┤labeled.png]

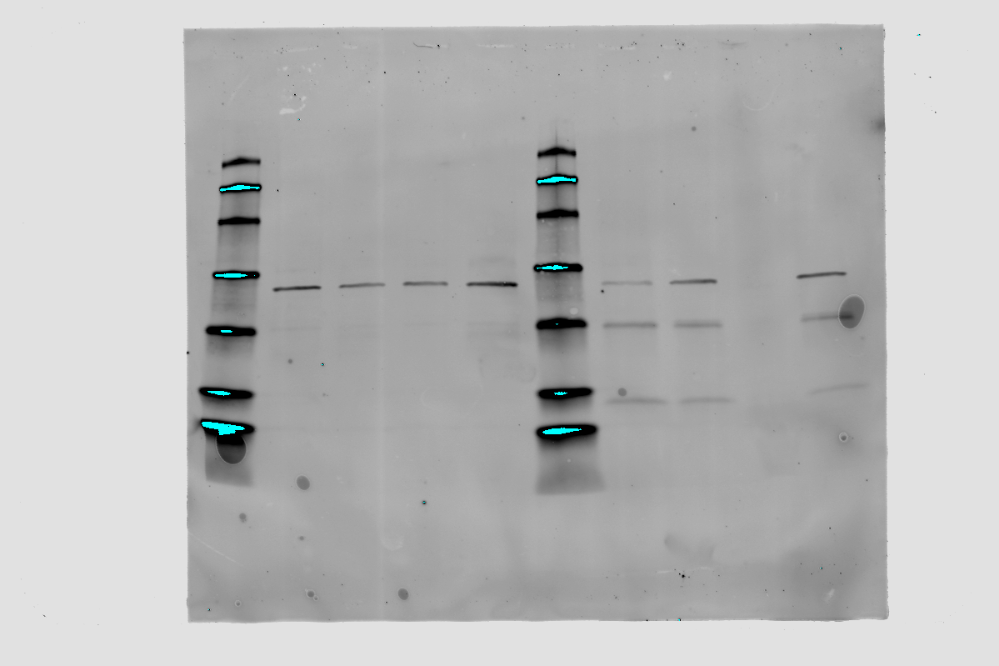

Supplement: Figure 2—source data 1. [file elife-97027-fig2-data1.zip › Figure 2 - Source data 1/Figure 2 - 2D raw 2.tif]

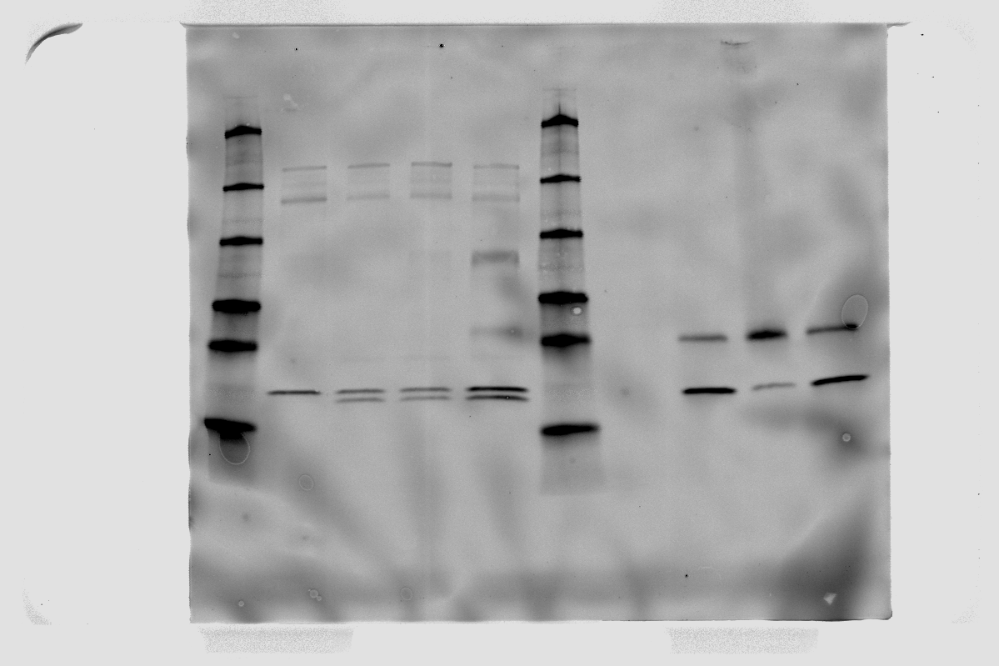

Supplement: Figure 2—source data 1. [file elife-97027-fig2-data1.zip › Figure 2 - Source data 1/Figure 2 - 2D raw.tif]

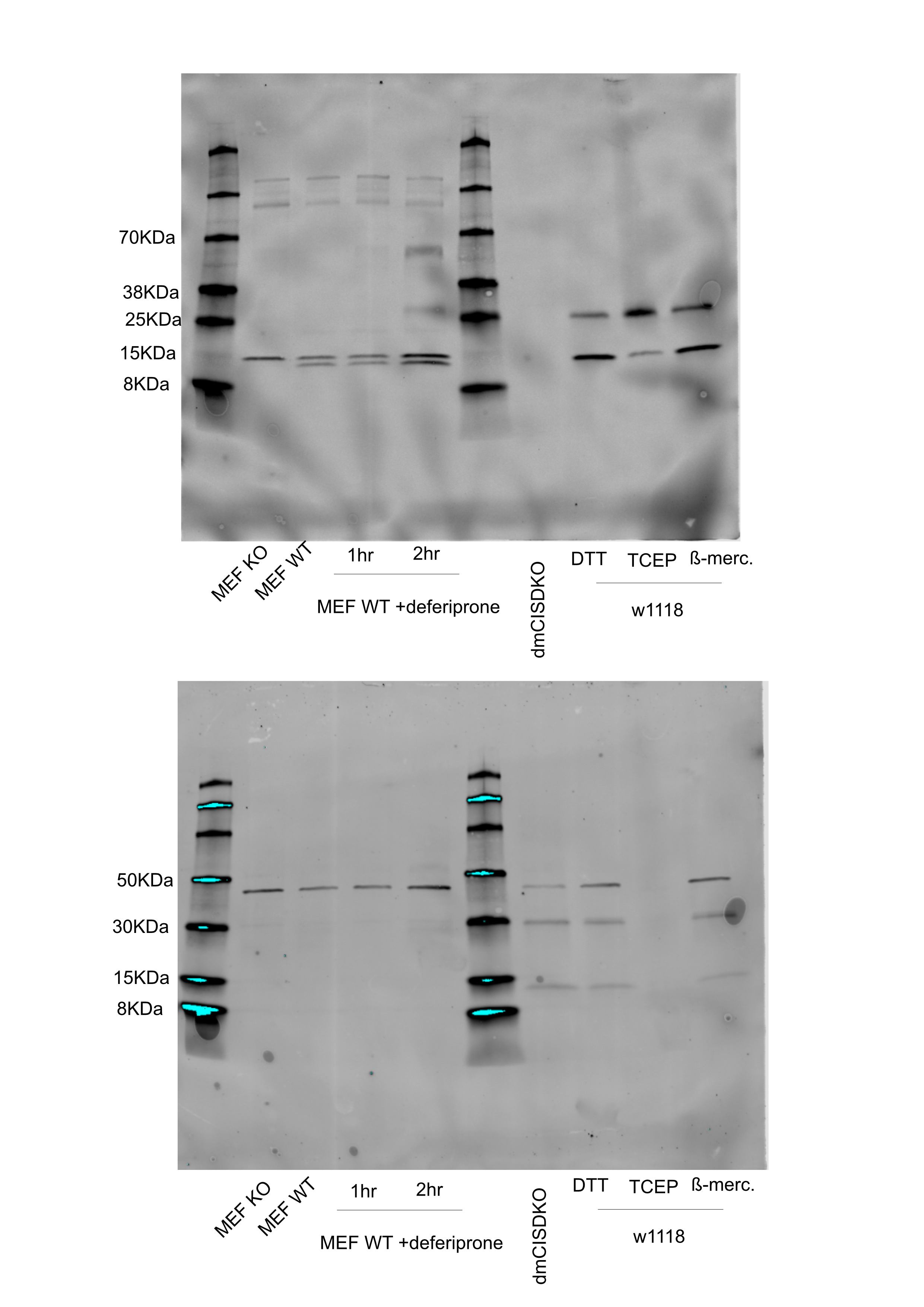

Supplement: Figure 2—source data 1. [file elife-97027-fig2-data1.zip › Figure 2 - Source data 1/Figure 2 - 2D labeled.png]

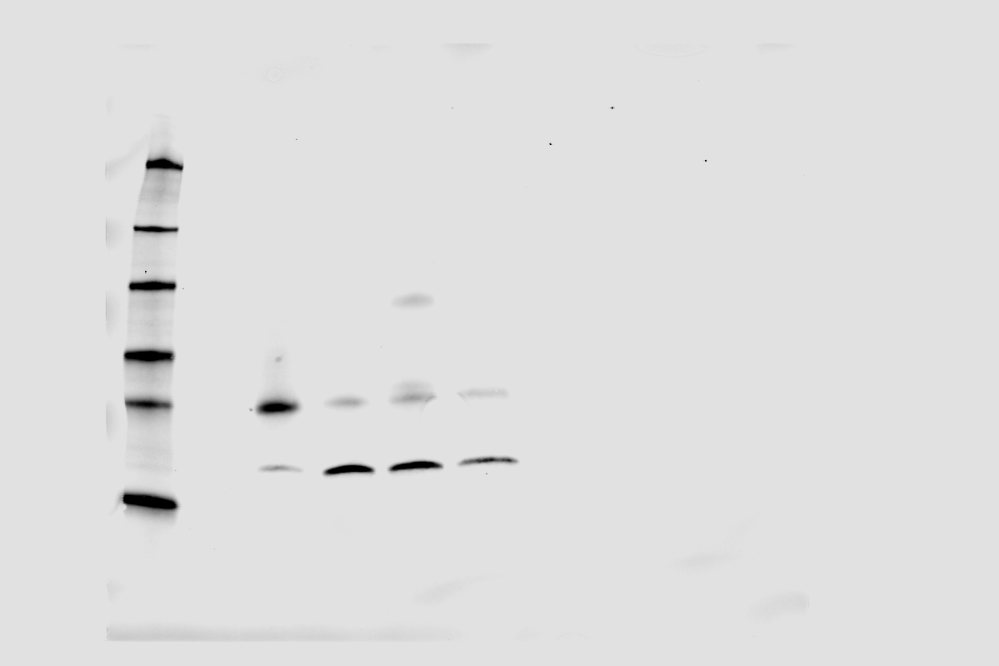

Supplement: Figure 3—source data 1. [file elife-97027-fig3-data1.zip › Figure 3 - Source data 1/Figure 3 - 3E- raw.tif]

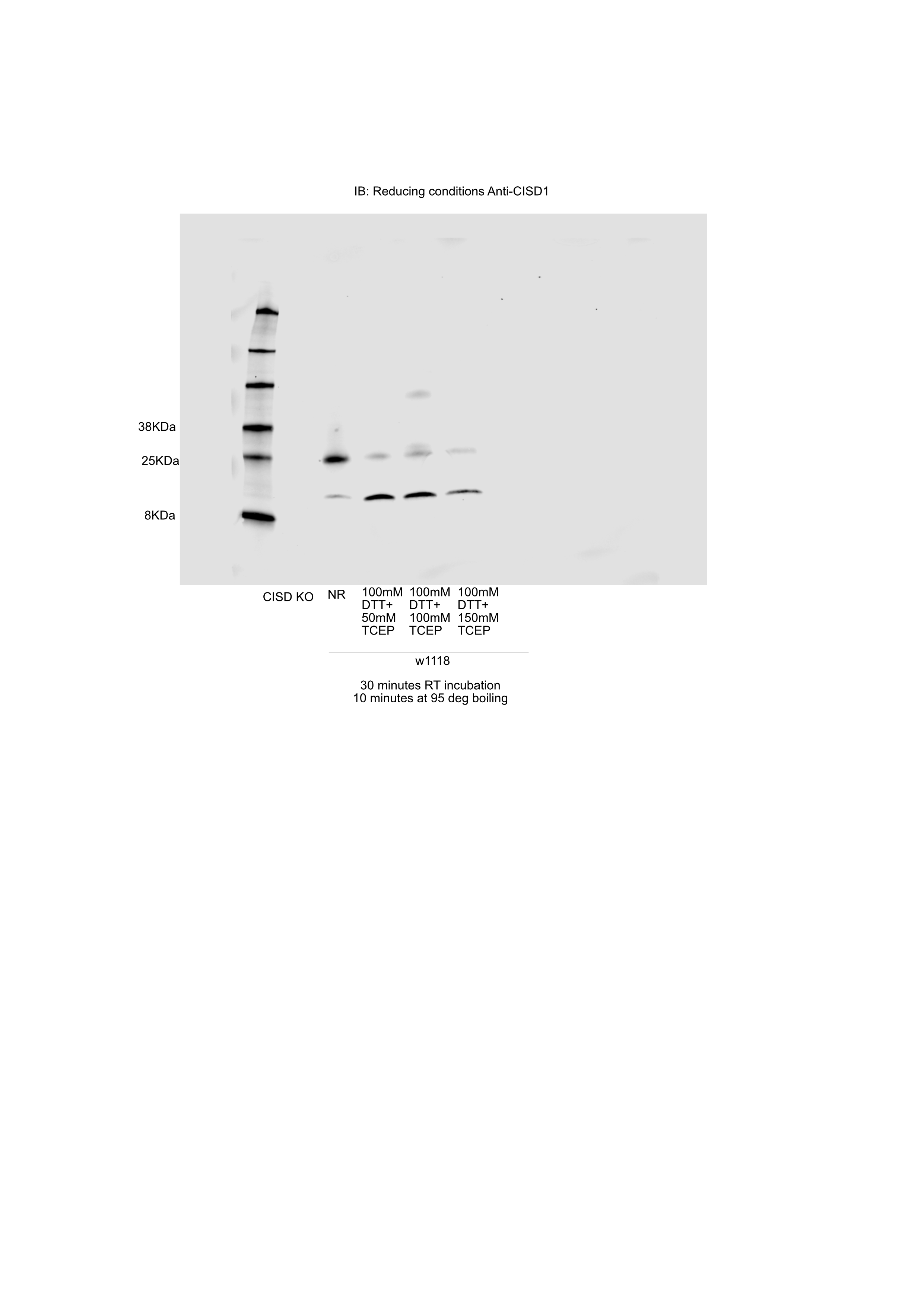

Supplement: Figure 3—source data 1. [file elife-97027-fig3-data1.zip › Figure 3 - Source data 1/Figure 3 - 3E- labeled.png]

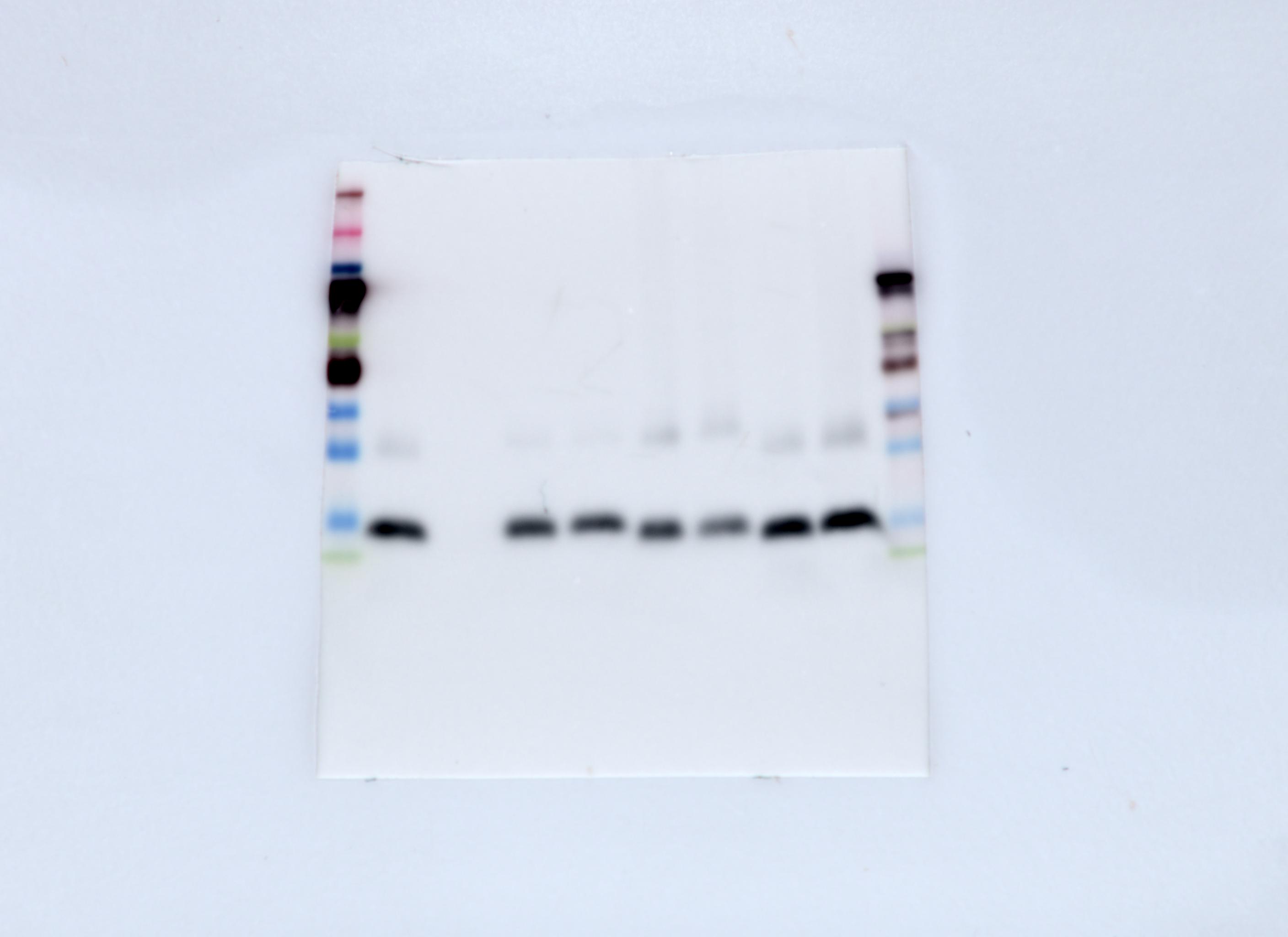

Supplement: Figure 3—source data 1. [file elife-97027-fig3-data1.zip › Figure 3 - Source data 1/Figure 3 - 3F - raw 1.jpg]

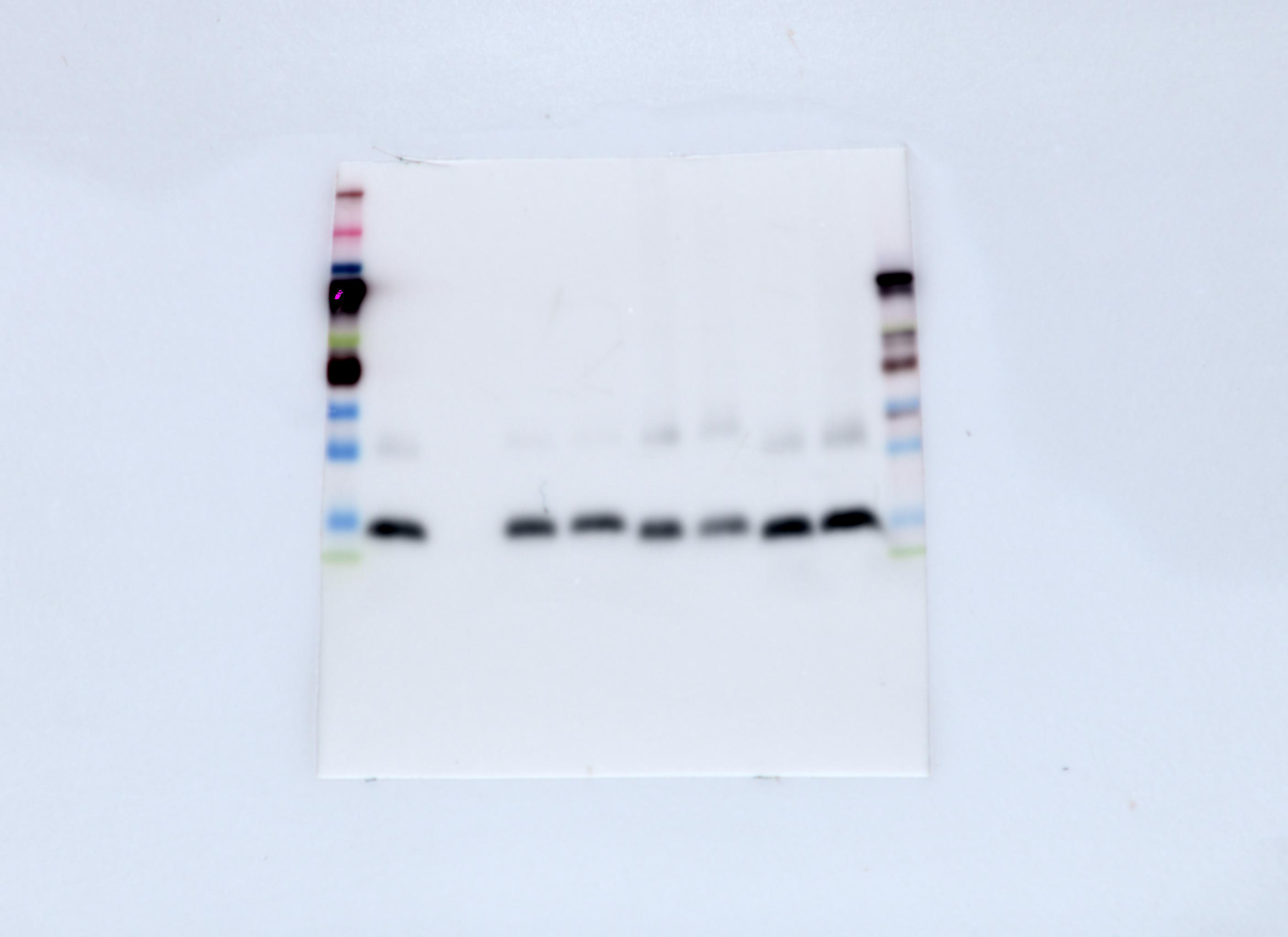

Supplement: Figure 3—source data 1. [file elife-97027-fig3-data1.zip › Figure 3 - Source data 1/Figure 3 - 3F - raw 2.jpg]

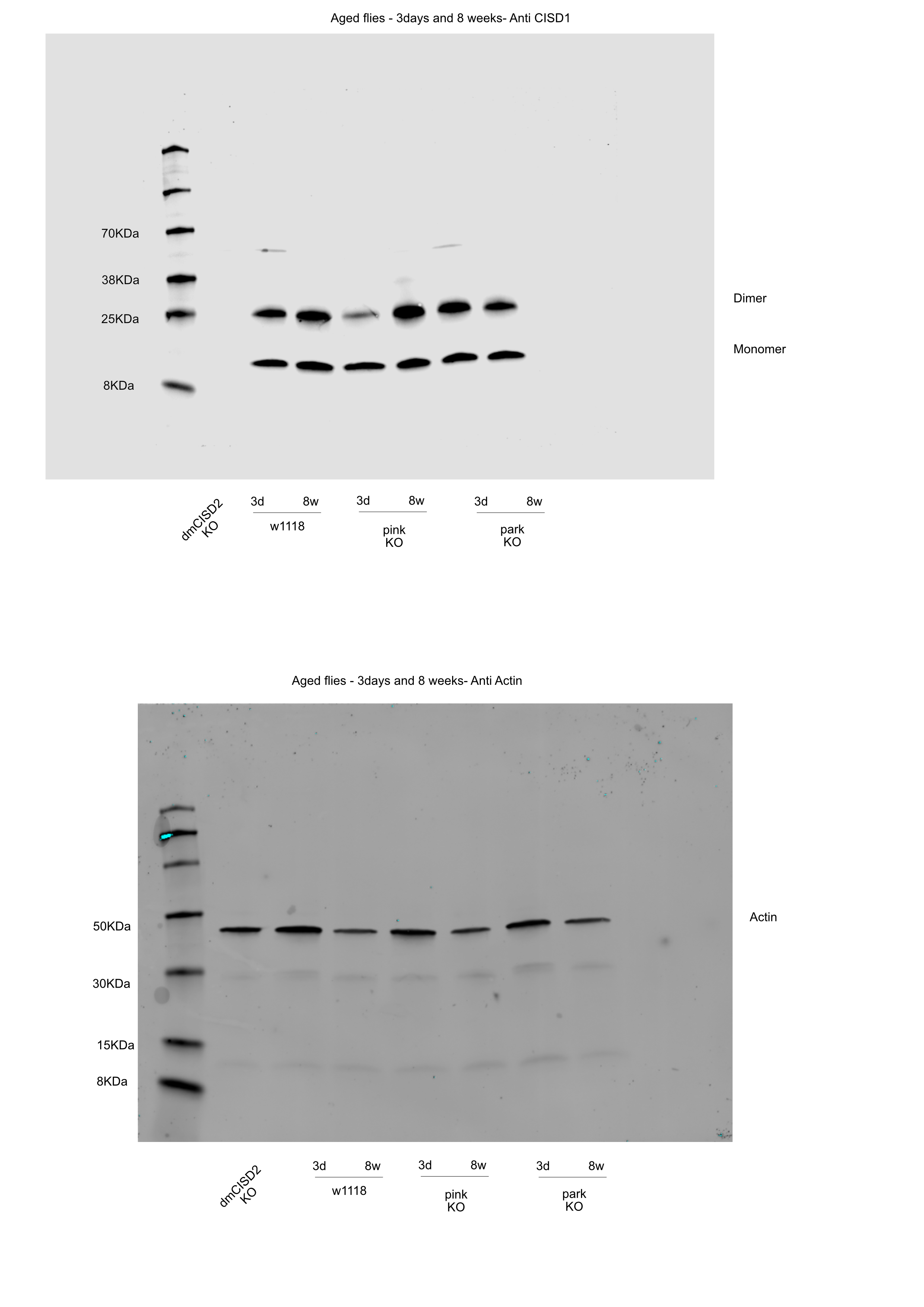

Supplement: Figure 3—source data 1. [file elife-97027-fig3-data1.zip › Figure 3 - Source data 1/Figure 3 - 3B - labeled.png]

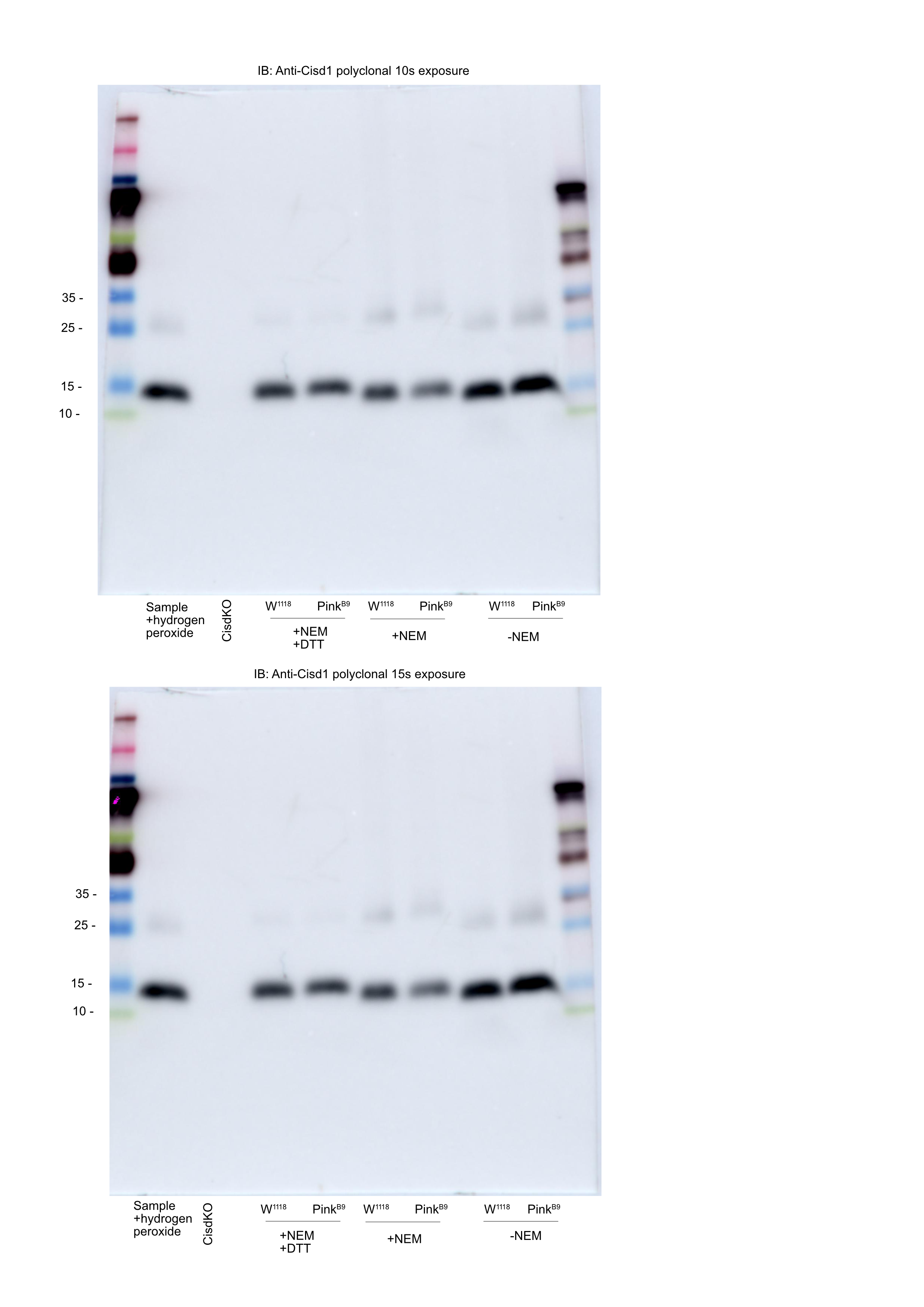

Supplement: Figure 3—source data 1. [file elife-97027-fig3-data1.zip › Figure 3 - Source data 1/Figure 3 - 3F - labeled.png]

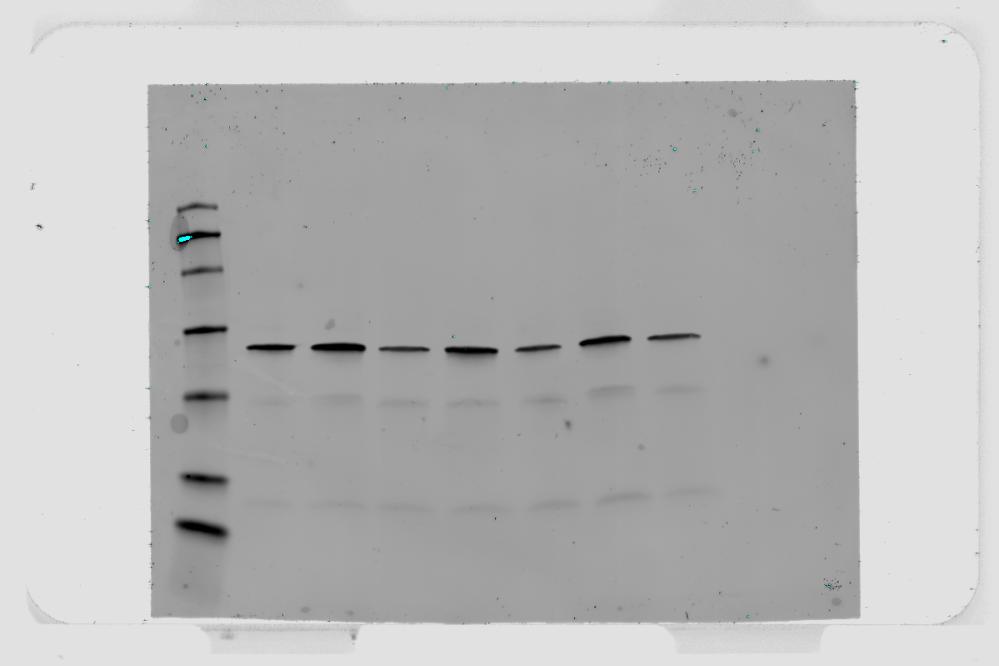

Supplement: Figure 3—source data 1. [file elife-97027-fig3-data1.zip › Figure 3 - Source data 1/Figure 3 - 3B - raw 1.tif]

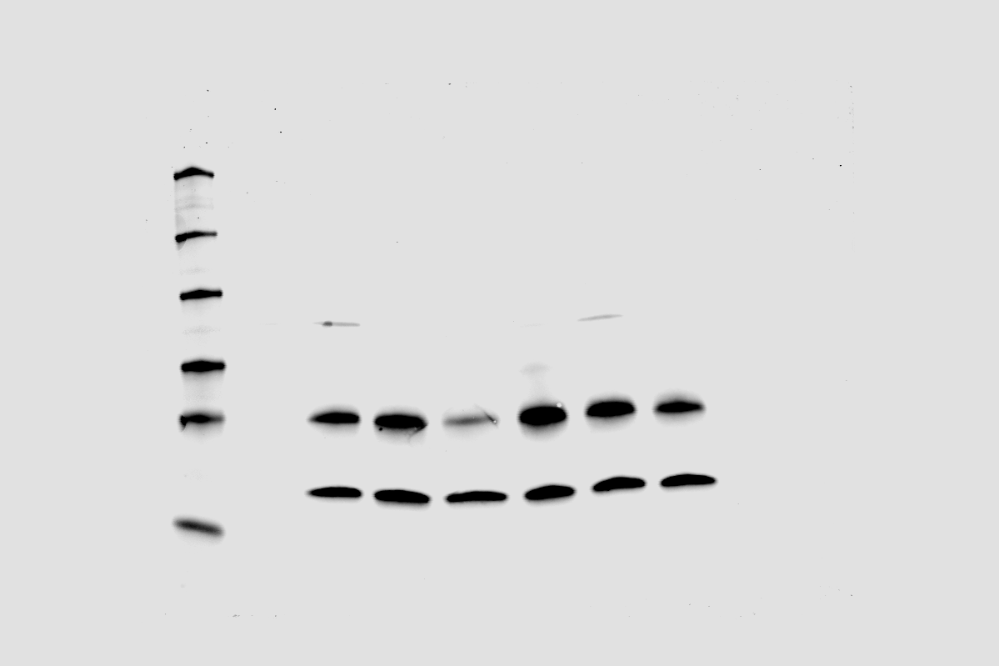

Supplement: Figure 3—source data 1. [file elife-97027-fig3-data1.zip › Figure 3 - Source data 1/Figure 3 - 3B - raw 2.tif]

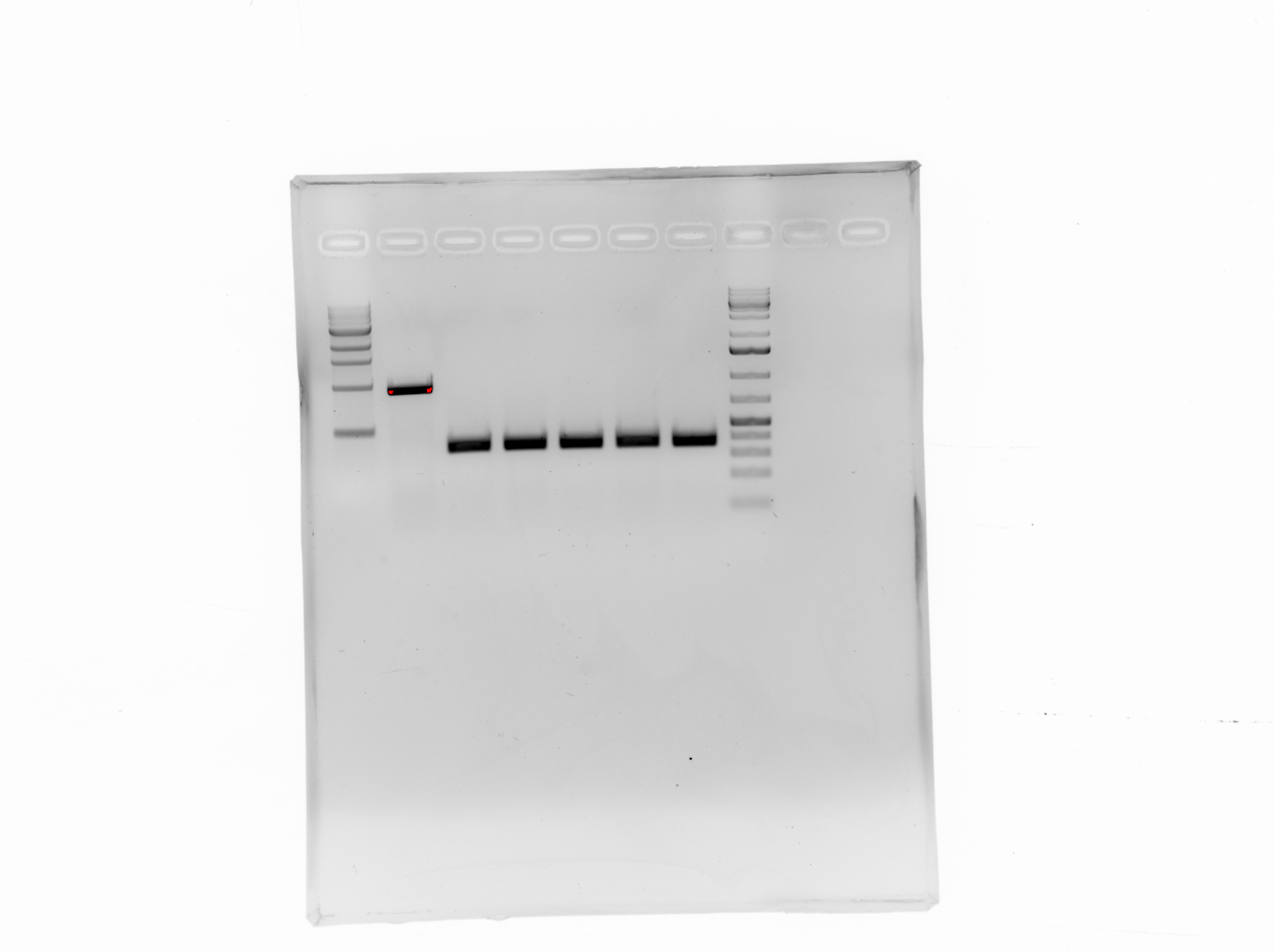

Supplement: Figure 3—figure supplement 1—source data 1. [file elife-97027-fig3-figsupp1-data1.zip › Figure 3 - figure supplement 1 - source data 1/Figure 3 - figure supplement 1 - raw 2 .tif]

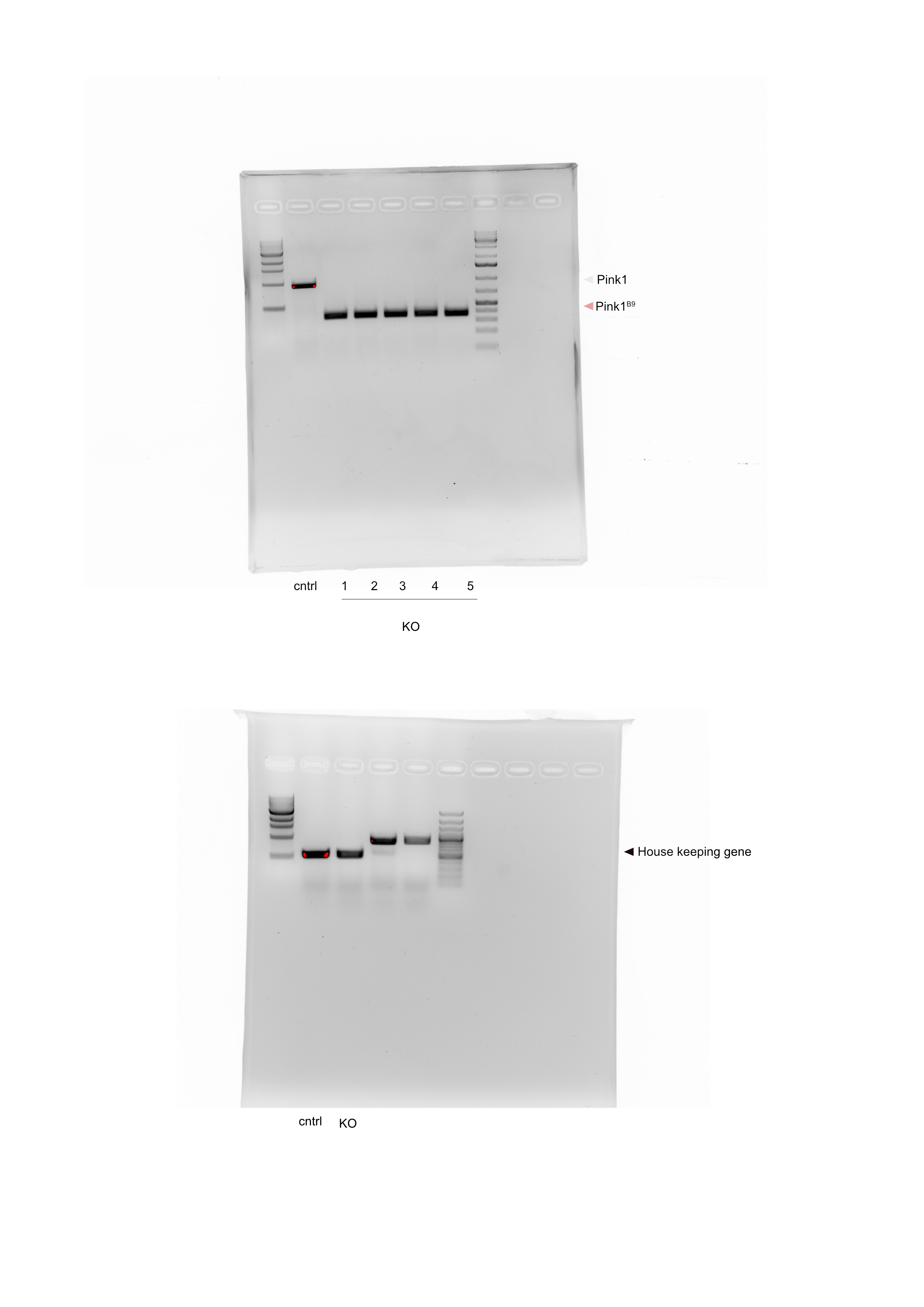

Supplement: Figure 3—figure supplement 1—source data 1. [file elife-97027-fig3-figsupp1-data1.zip › Figure 3 - figure supplement 1 - source data 1/Figure 3 - figure supplement 1 - labeled.png]

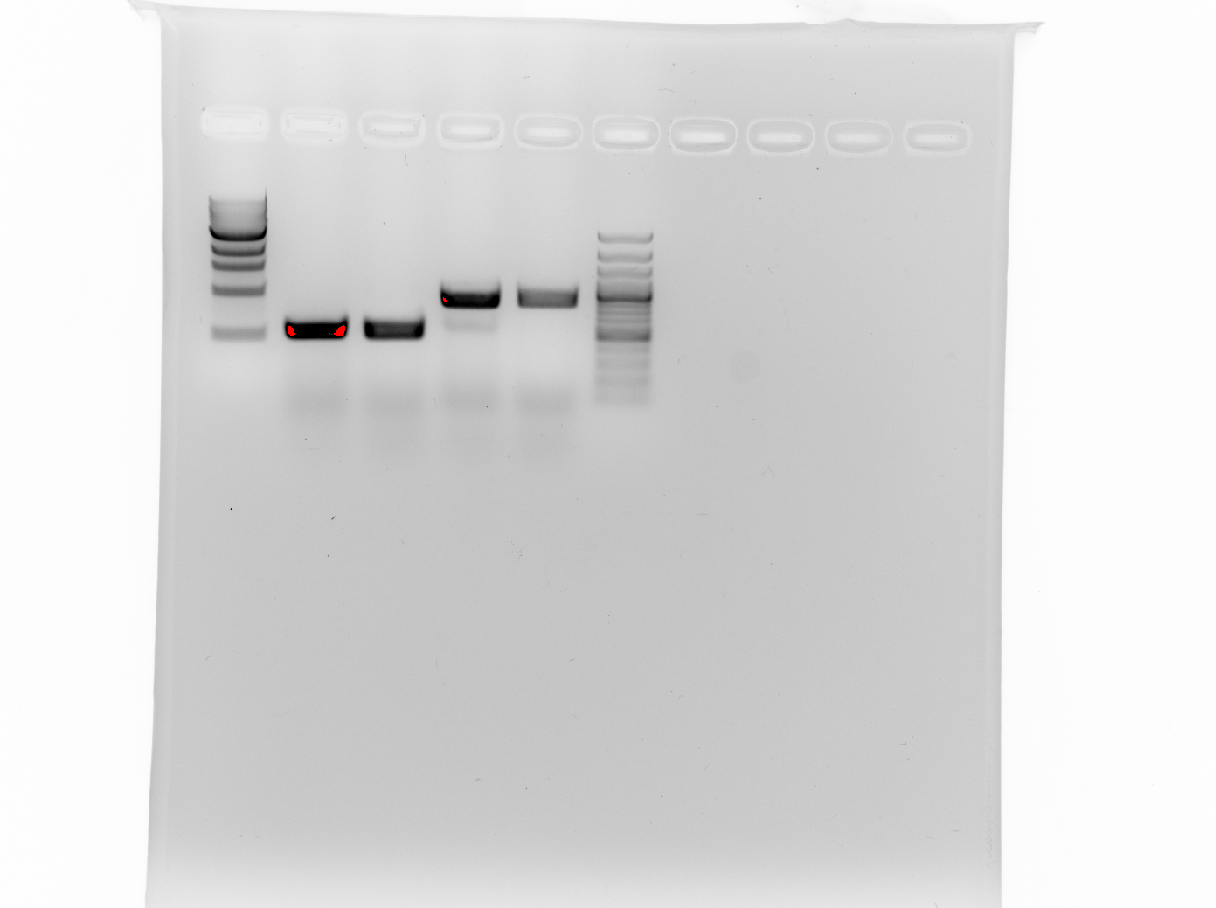

Supplement: Figure 3—figure supplement 1—source data 1. [file elife-97027-fig3-figsupp1-data1.zip › Figure 3 - figure supplement 1 - source data 1/Figure 3 - figure supplement 1 - raw.tif]

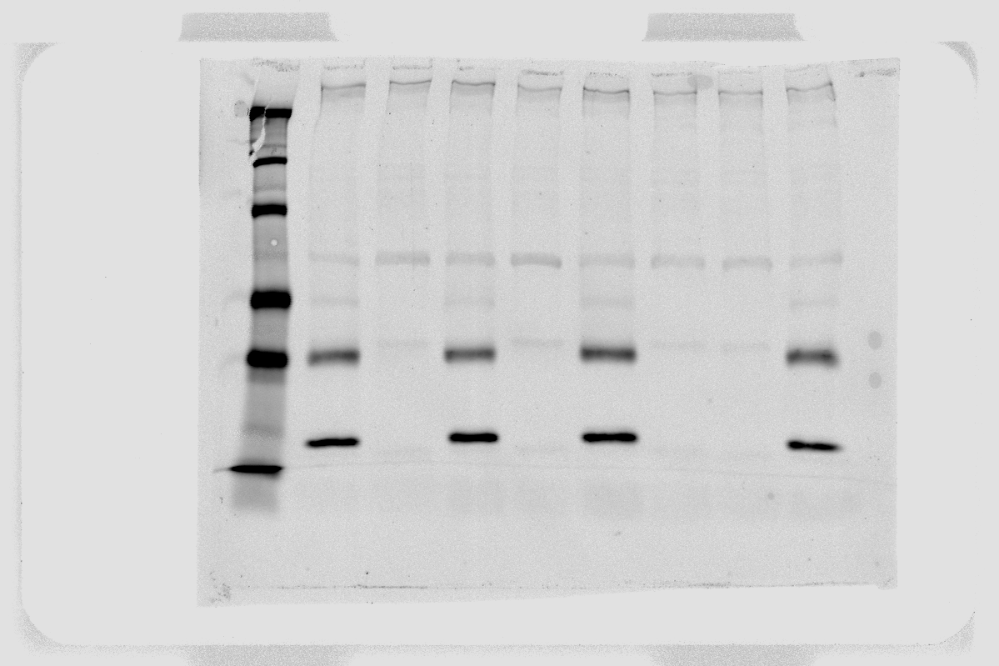

Supplement: Figure 5—source data 1. [file elife-97027-fig5-data1.zip › Figure 5 - Source data 1/Figure 5 - 5A - raw1.tif]

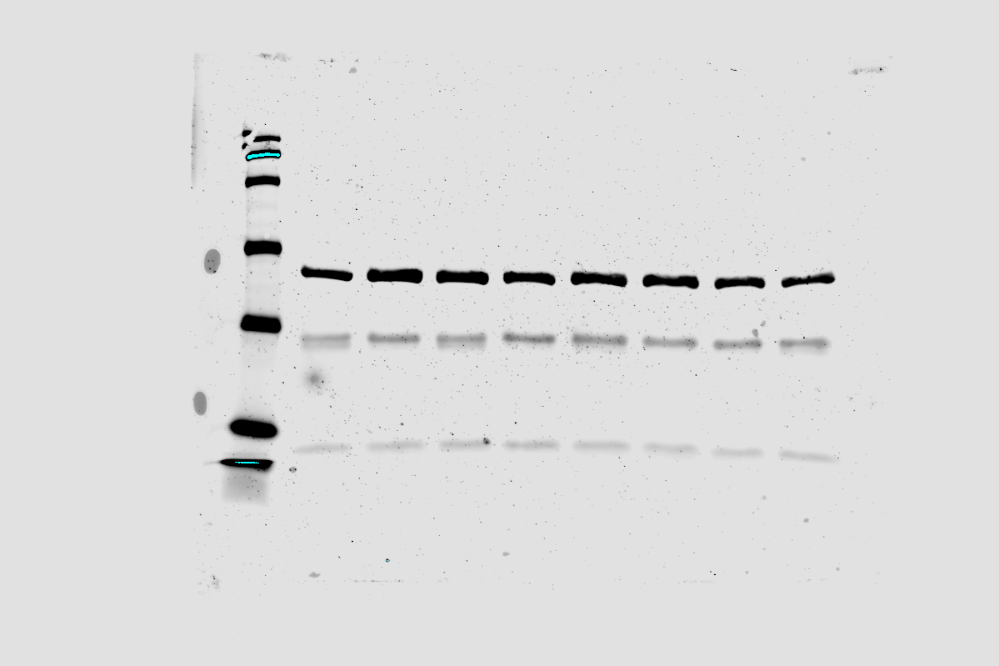

Supplement: Figure 5—source data 1. [file elife-97027-fig5-data1.zip › Figure 5 - Source data 1/Figure 5 - 5A - raw2.tif]

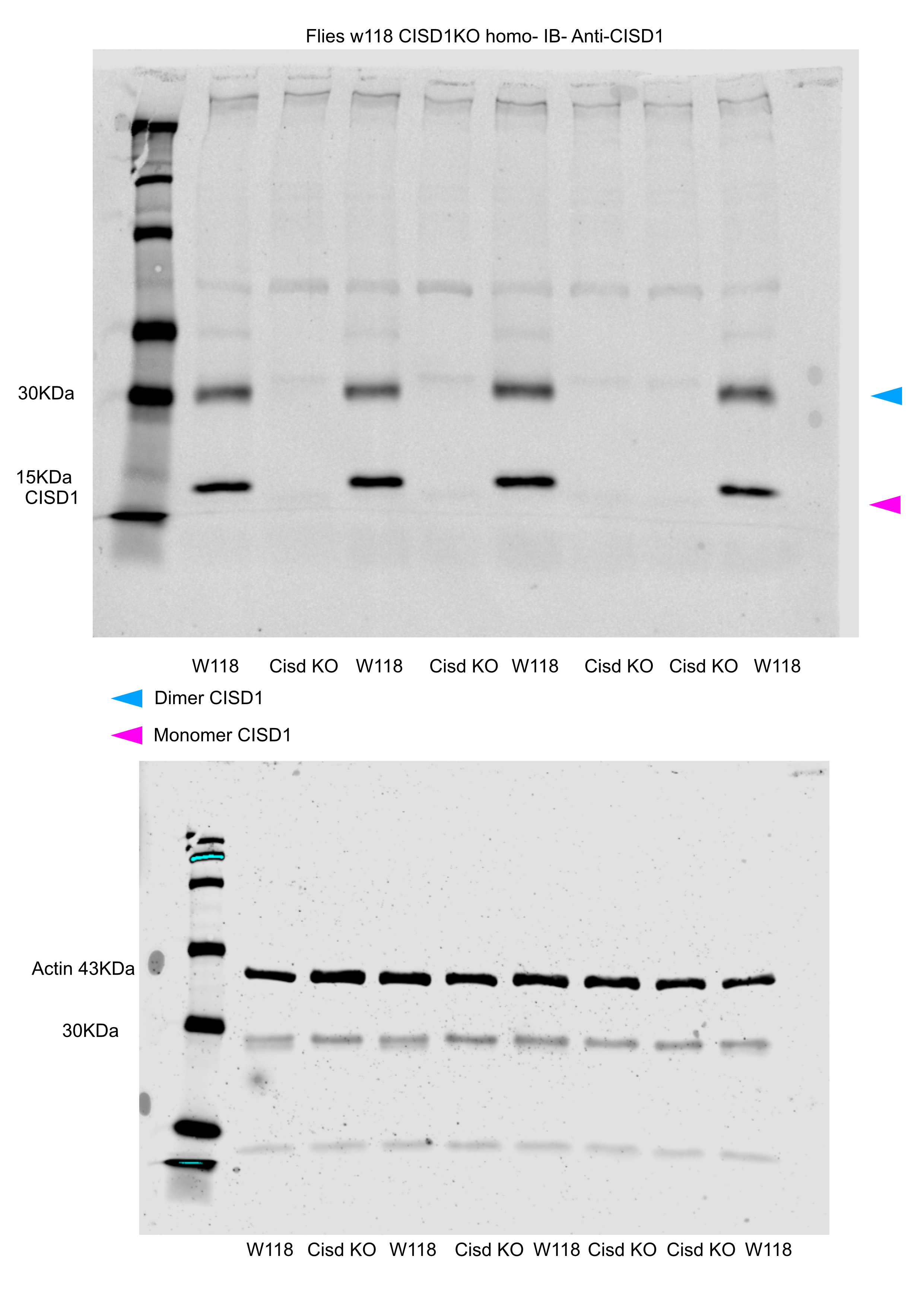

Supplement: Figure 5—source data 1. [file elife-97027-fig5-data1.zip › Figure 5 - Source data 1/Figure 5 - 5A- labeled.png]

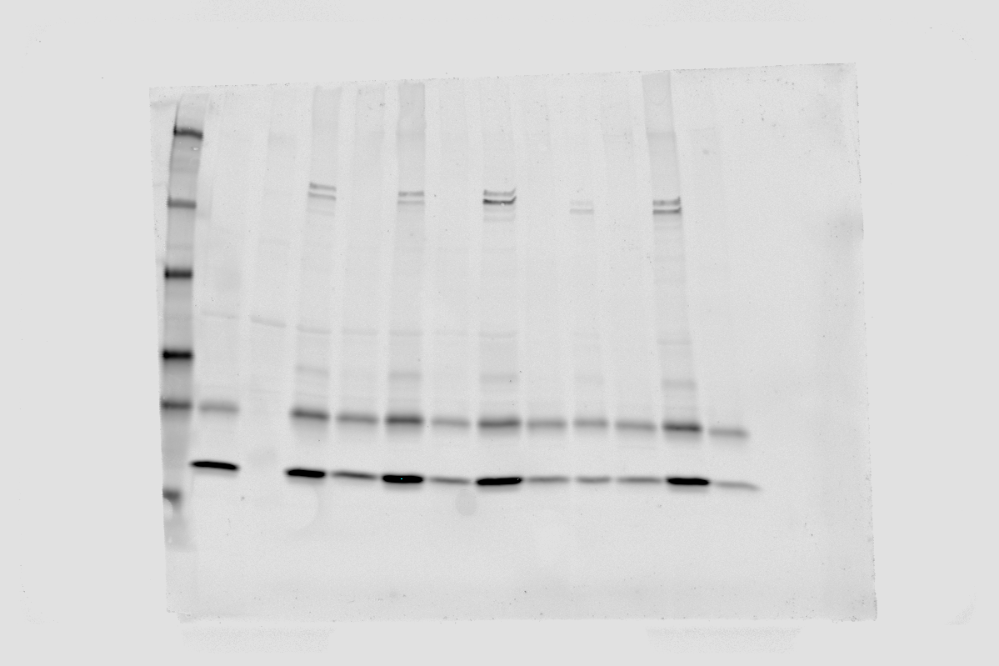

Supplement: Figure 5—figure supplement 1—source data 1. [file elife-97027-fig5-figsupp1-data1.zip › Figure 5 - figure supplement 1 - source data 1 /Figure 5 - figure supplement 1 - 1A- raw 2.tif]

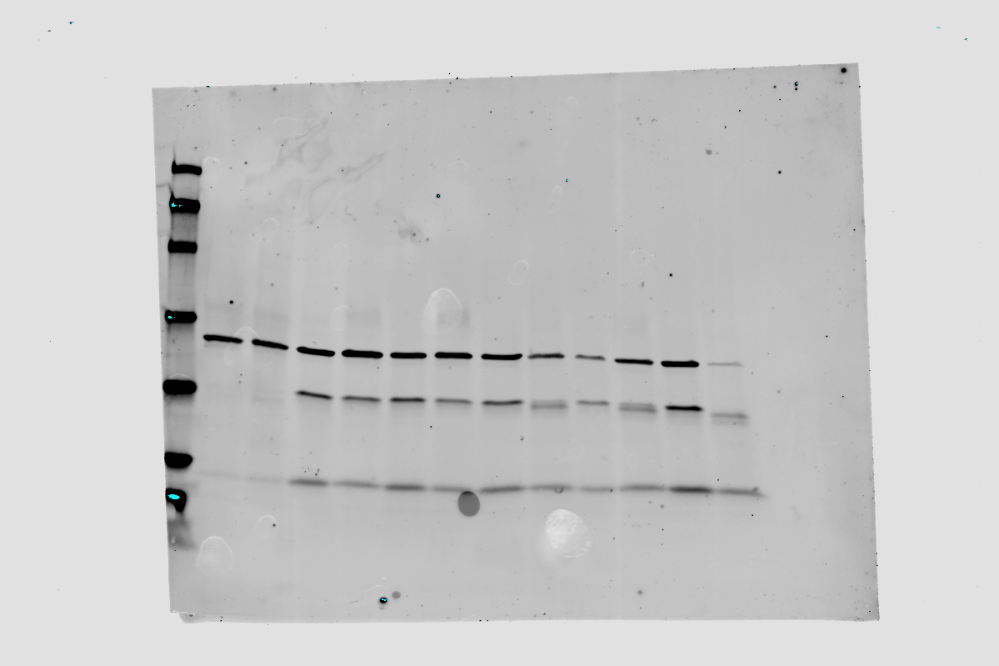

Supplement: Figure 5—figure supplement 1—source data 1. [file elife-97027-fig5-figsupp1-data1.zip › Figure 5 - figure supplement 1 - source data 1 /Figure 5 - figure supplement 1 - 1A- raw 1 .tif]

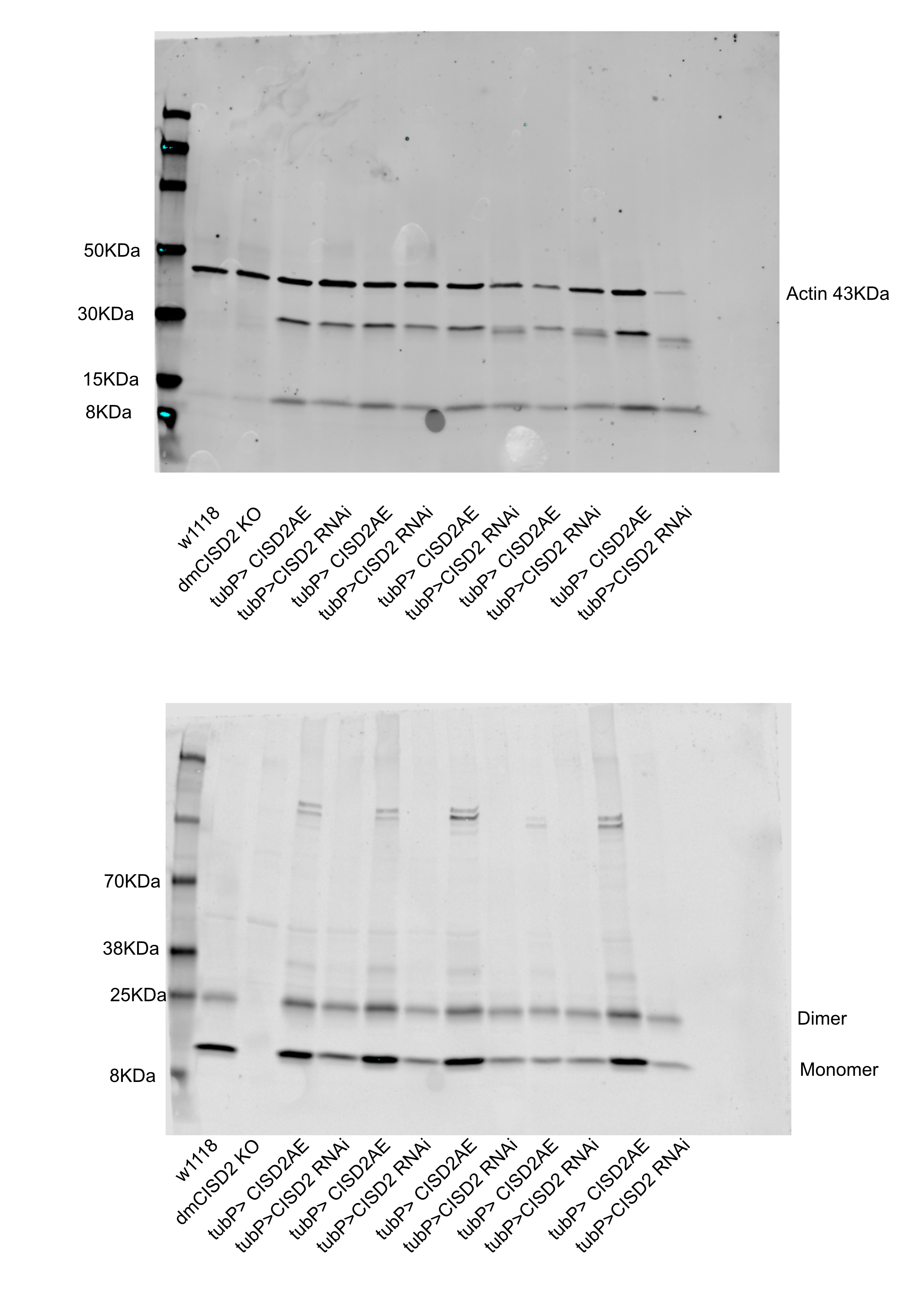

Supplement: Figure 5—figure supplement 1—source data 1. [file elife-97027-fig5-figsupp1-data1.zip › Figure 5 - figure supplement 1 - source data 1 /Figure 5 - figure supplement 1 - 1A- labeled.png]

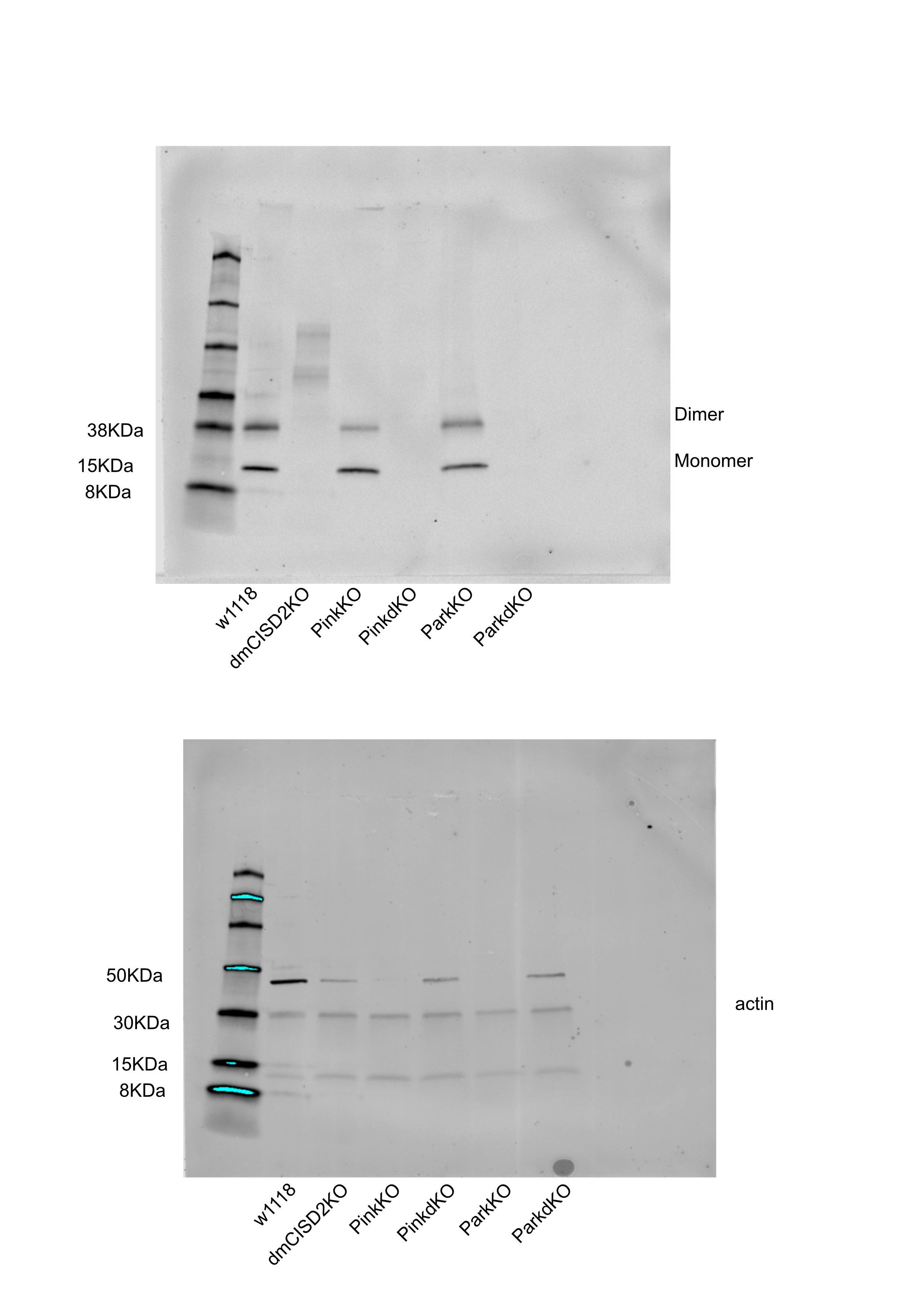

Supplement: Figure 5—figure supplement 3—source data 1. [file elife-97027-fig5-figsupp3-data1.zip › Figure 5 - figure supplement 3 -source data 1/Figure 5 - figure supplement 3 - labeled.png]

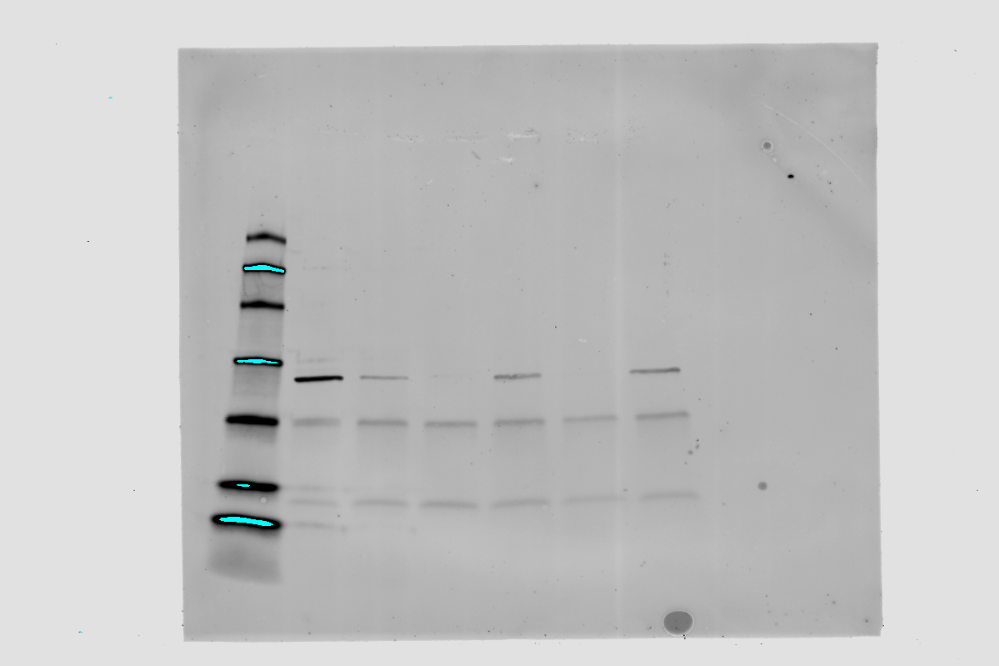

Supplement: Figure 5—figure supplement 3—source data 1. [file elife-97027-fig5-figsupp3-data1.zip › Figure 5 - figure supplement 3 -source data 1/Figure 5 - figure supplement 3 - raw 1.tif]

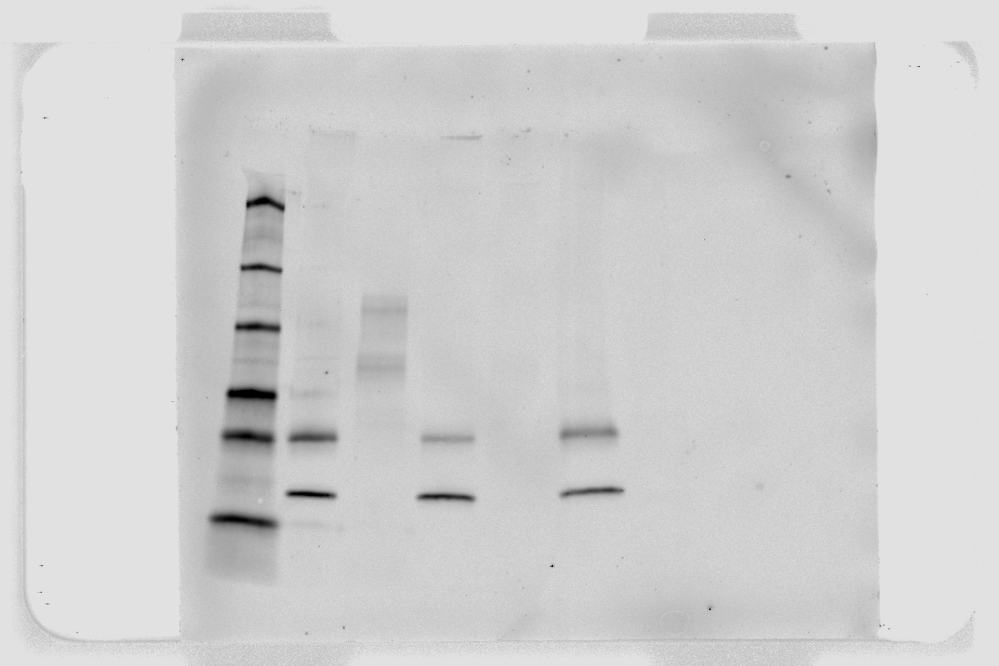

Supplement: Figure 5—figure supplement 3—source data 1. [file elife-97027-fig5-figsupp3-data1.zip › Figure 5 - figure supplement 3 -source data 1/Figure 5 - figure supplement 3 - raw 2.tif]

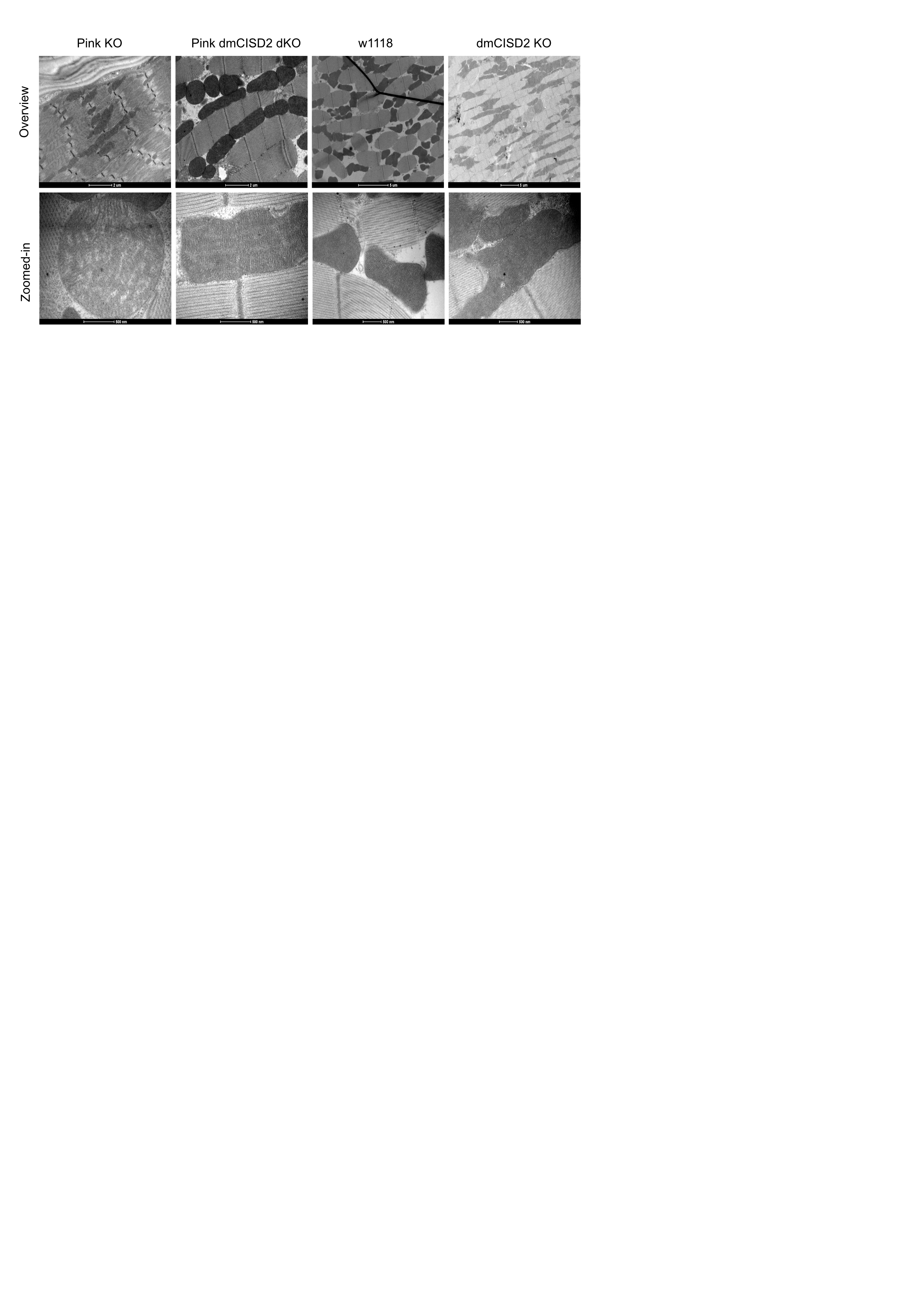

Supplement: Figure 5—figure supplement 4—source data 1. [file elife-97027-fig5-figsupp4-data1.zip › Figure 5 - figure supplement 4 - source data 1/Figure 5 - figure supplement 4 .png]

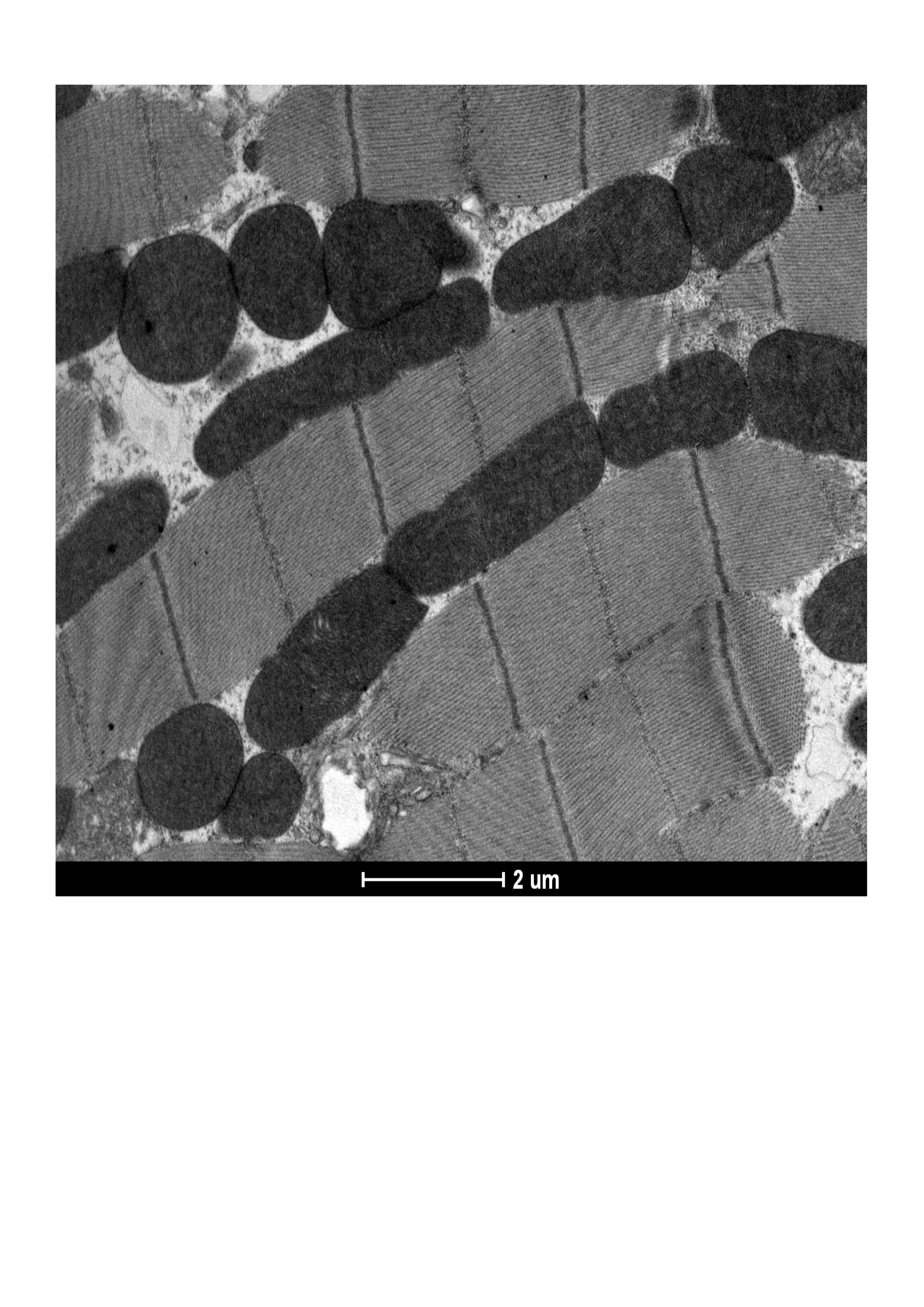

Supplement: Figure 5—figure supplement 4—source data 2. [file elife-97027-fig5-figsupp4-data2.zip › Figure 5 - figure supplement 4 - source data 2/DKO overview.png]

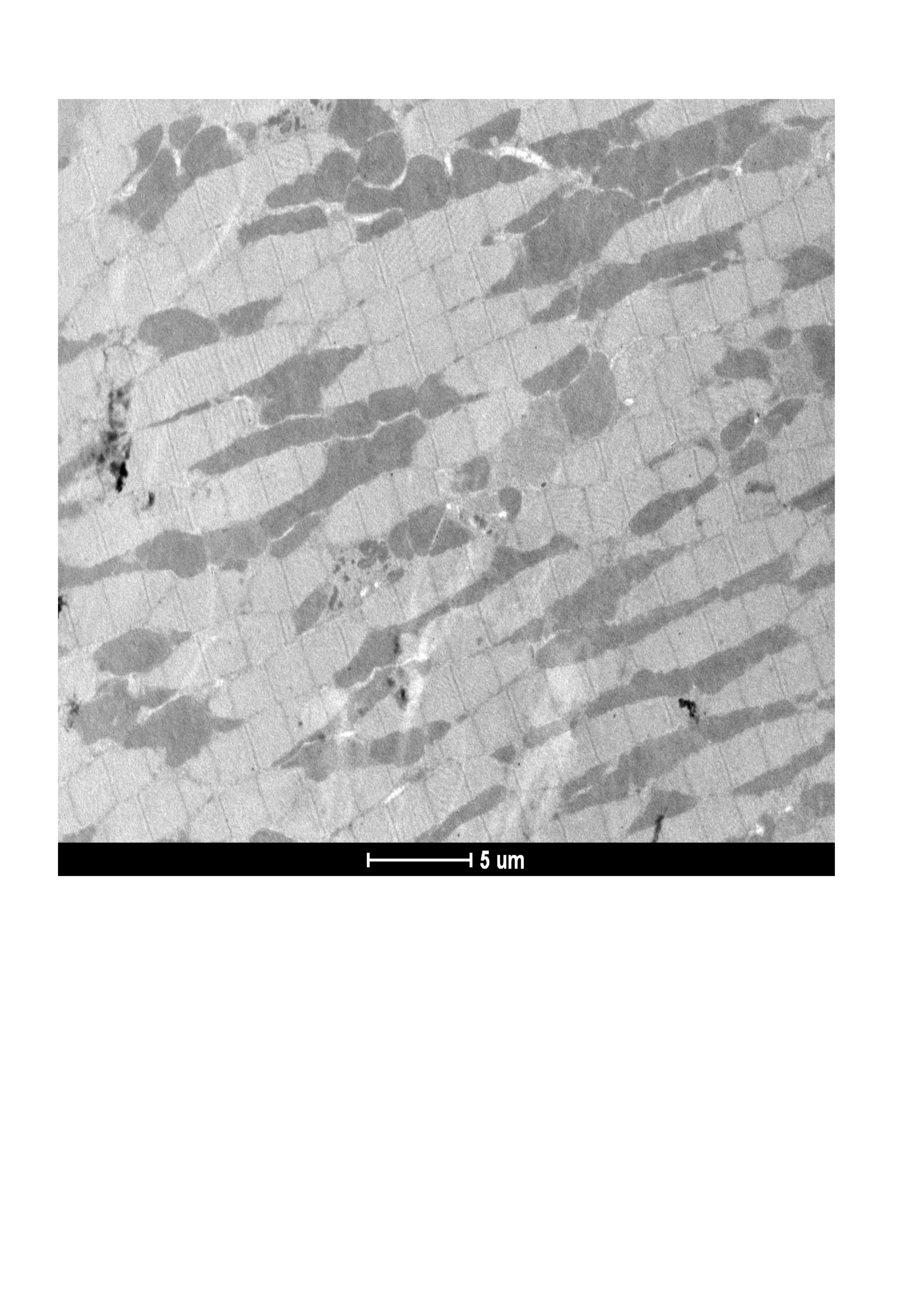

Supplement: Figure 5—figure supplement 4—source data 2. [file elife-97027-fig5-figsupp4-data2.zip › Figure 5 - figure supplement 4 - source data 2/Cisd KO overview.png]

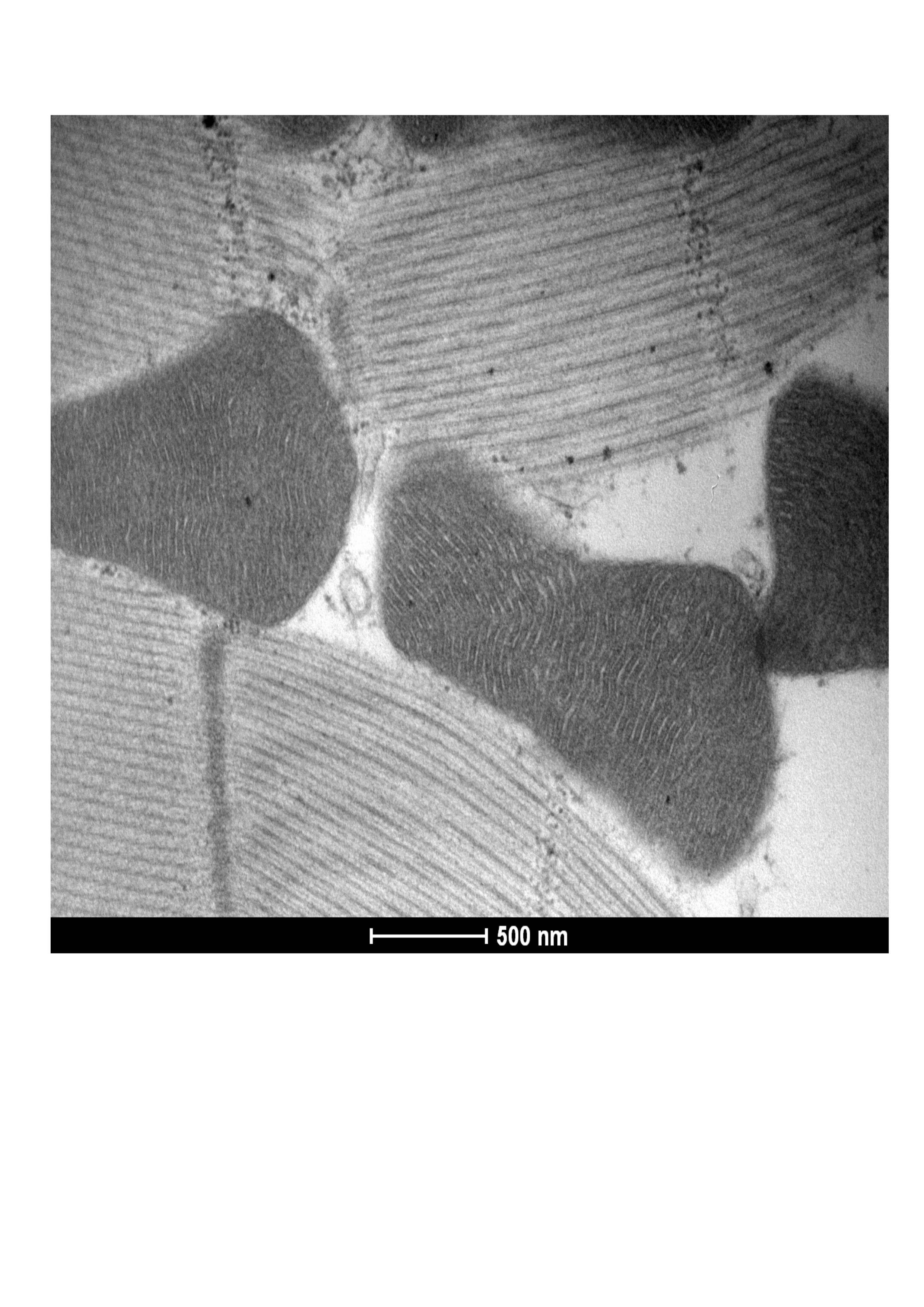

Supplement: Figure 5—figure supplement 4—source data 2. [file elife-97027-fig5-figsupp4-data2.zip › Figure 5 - figure supplement 4 - source data 2/w1118 zoomed in.png]

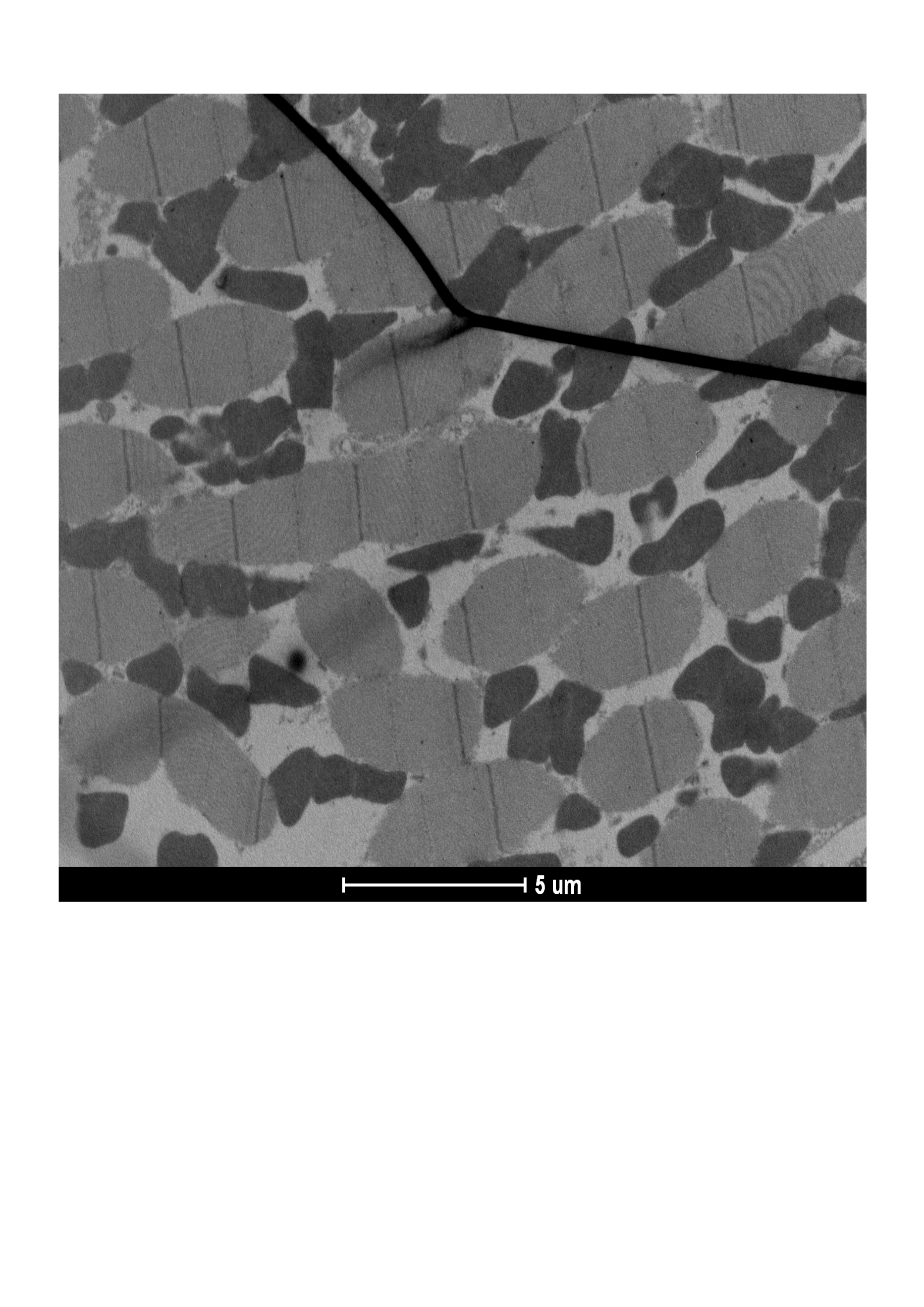

Supplement: Figure 5—figure supplement 4—source data 2. [file elife-97027-fig5-figsupp4-data2.zip › Figure 5 - figure supplement 4 - source data 2/w1118 overview.png]

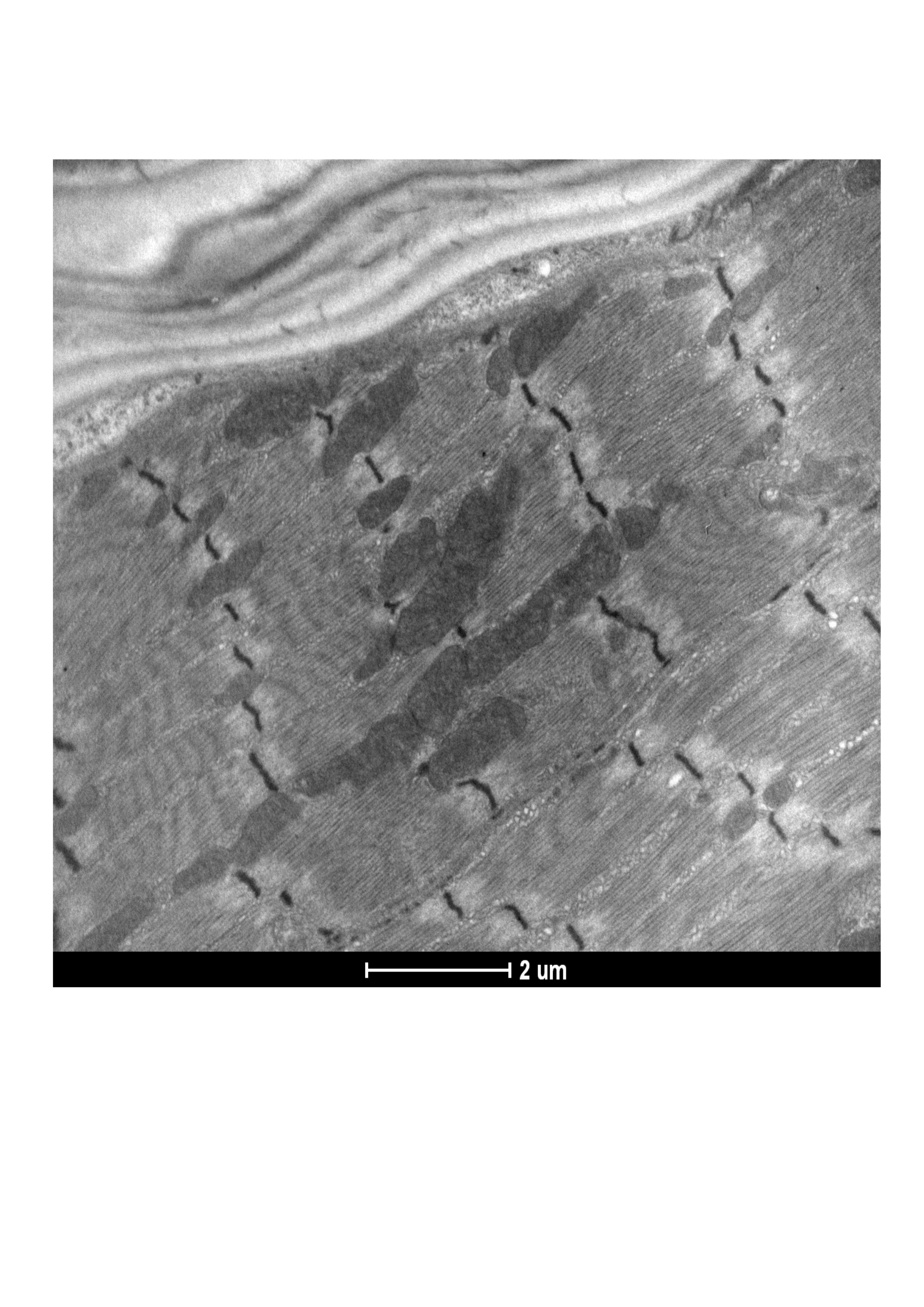

Supplement: Figure 5—figure supplement 4—source data 2. [file elife-97027-fig5-figsupp4-data2.zip › Figure 5 - figure supplement 4 - source data 2/Pink1B9 overview.png]

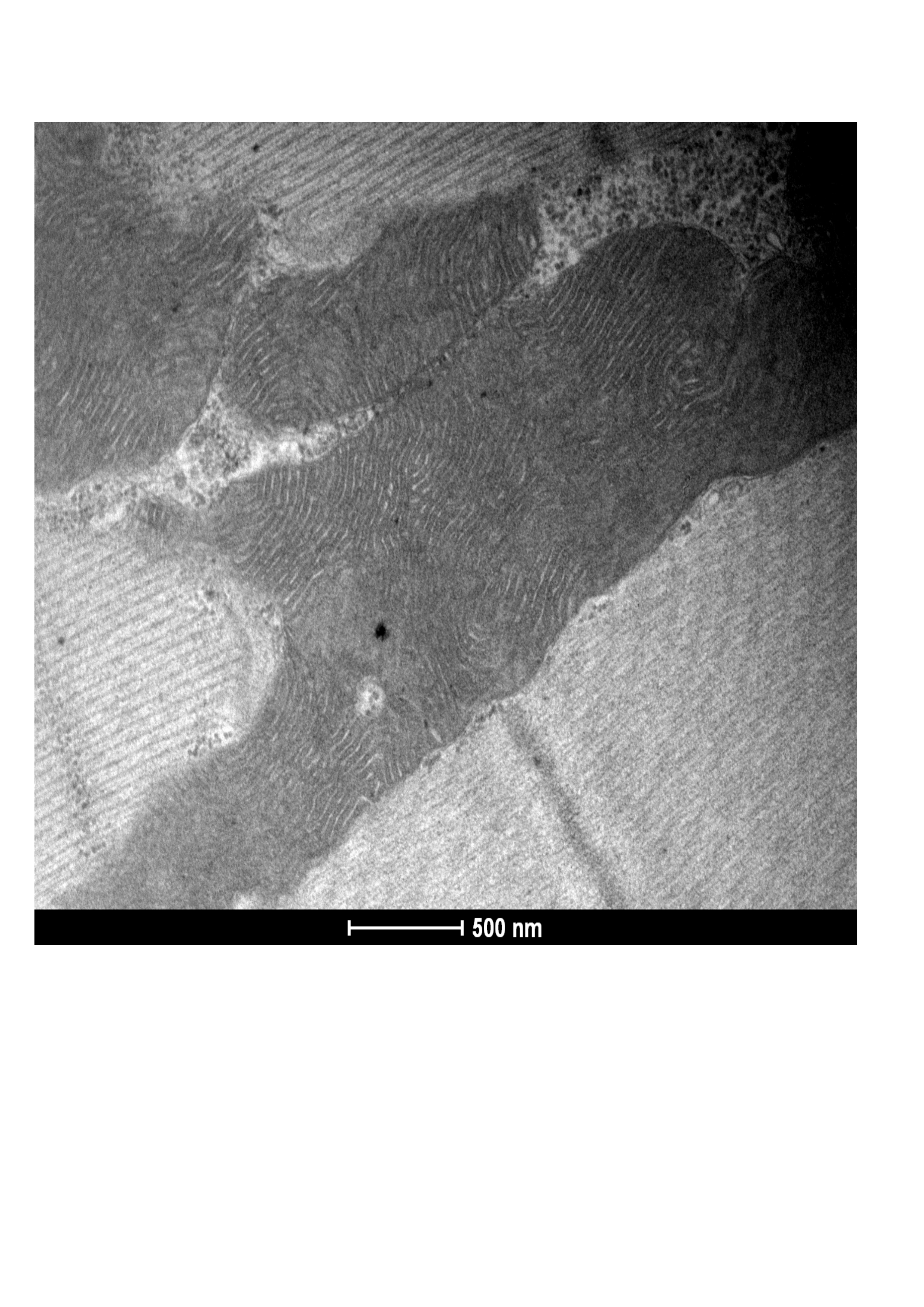

Supplement: Figure 5—figure supplement 4—source data 2. [file elife-97027-fig5-figsupp4-data2.zip › Figure 5 - figure supplement 4 - source data 2/Cisd KO zooomed in.png]

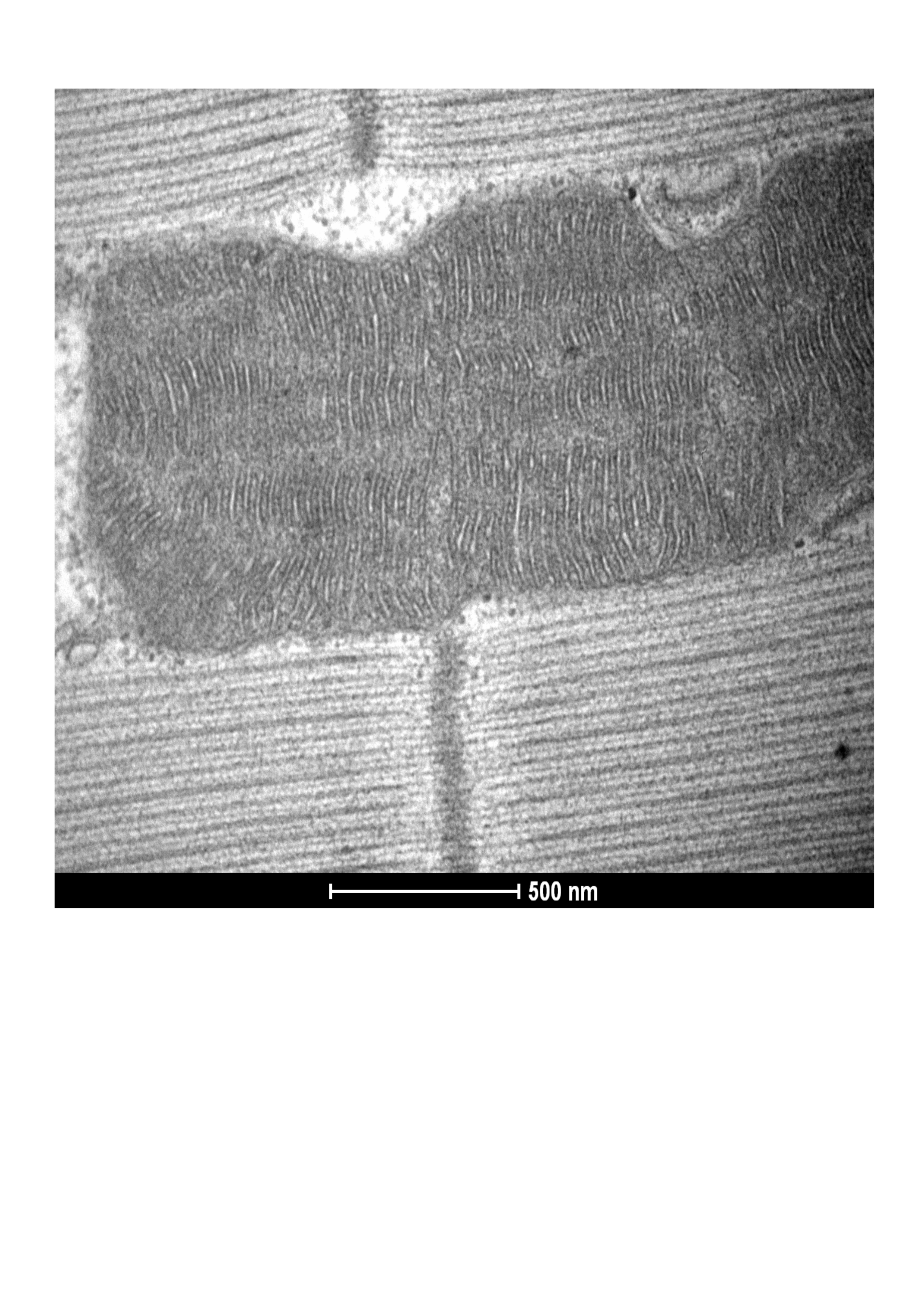

Supplement: Figure 5—figure supplement 4—source data 2. [file elife-97027-fig5-figsupp4-data2.zip › Figure 5 - figure supplement 4 - source data 2/DKO zoomed in.png]

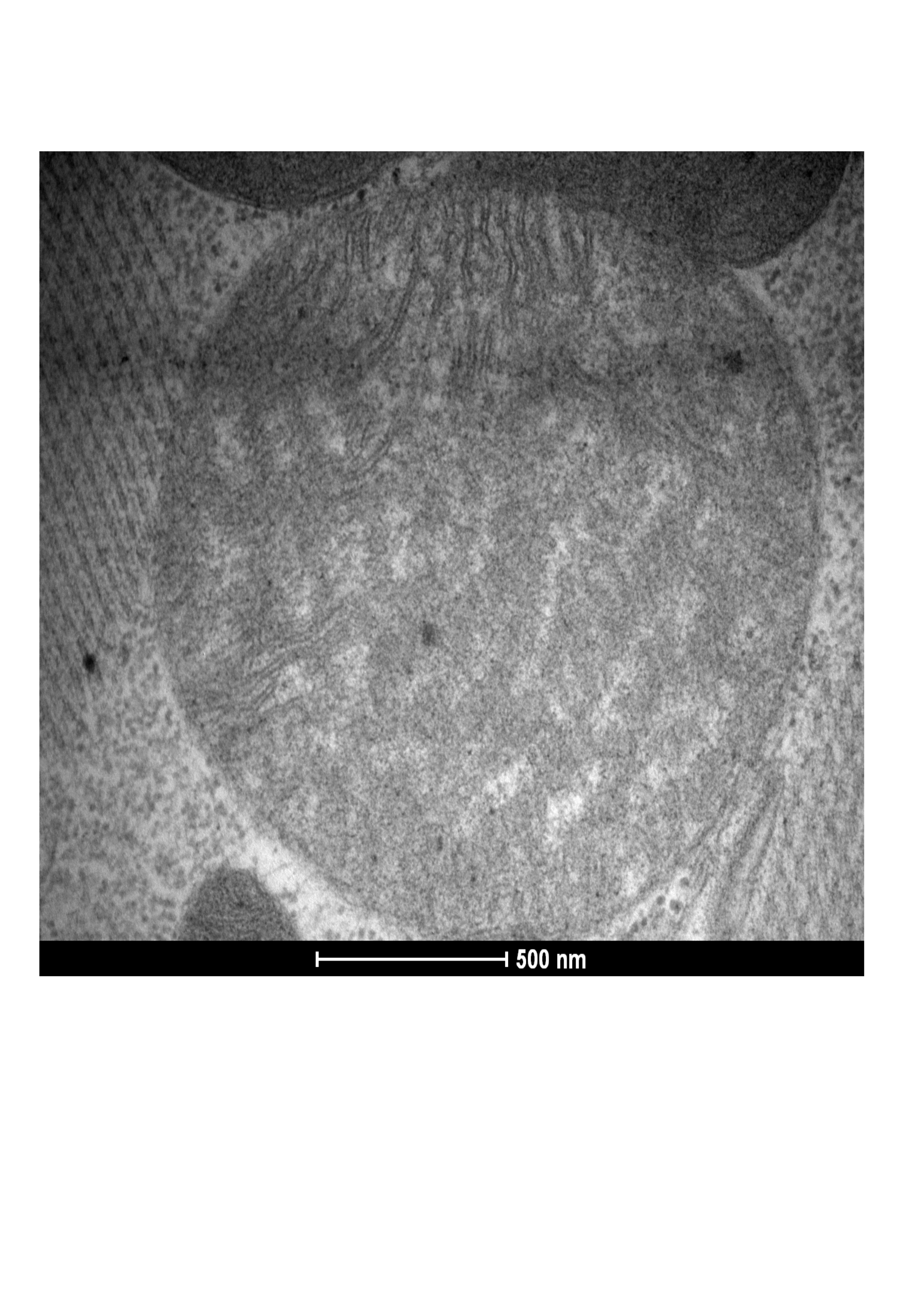

Supplement: Figure 5—figure supplement 4—source data 2. [file elife-97027-fig5-figsupp4-data2.zip › Figure 5 - figure supplement 4 - source data 2/Pink1B9 zoomed in.png]

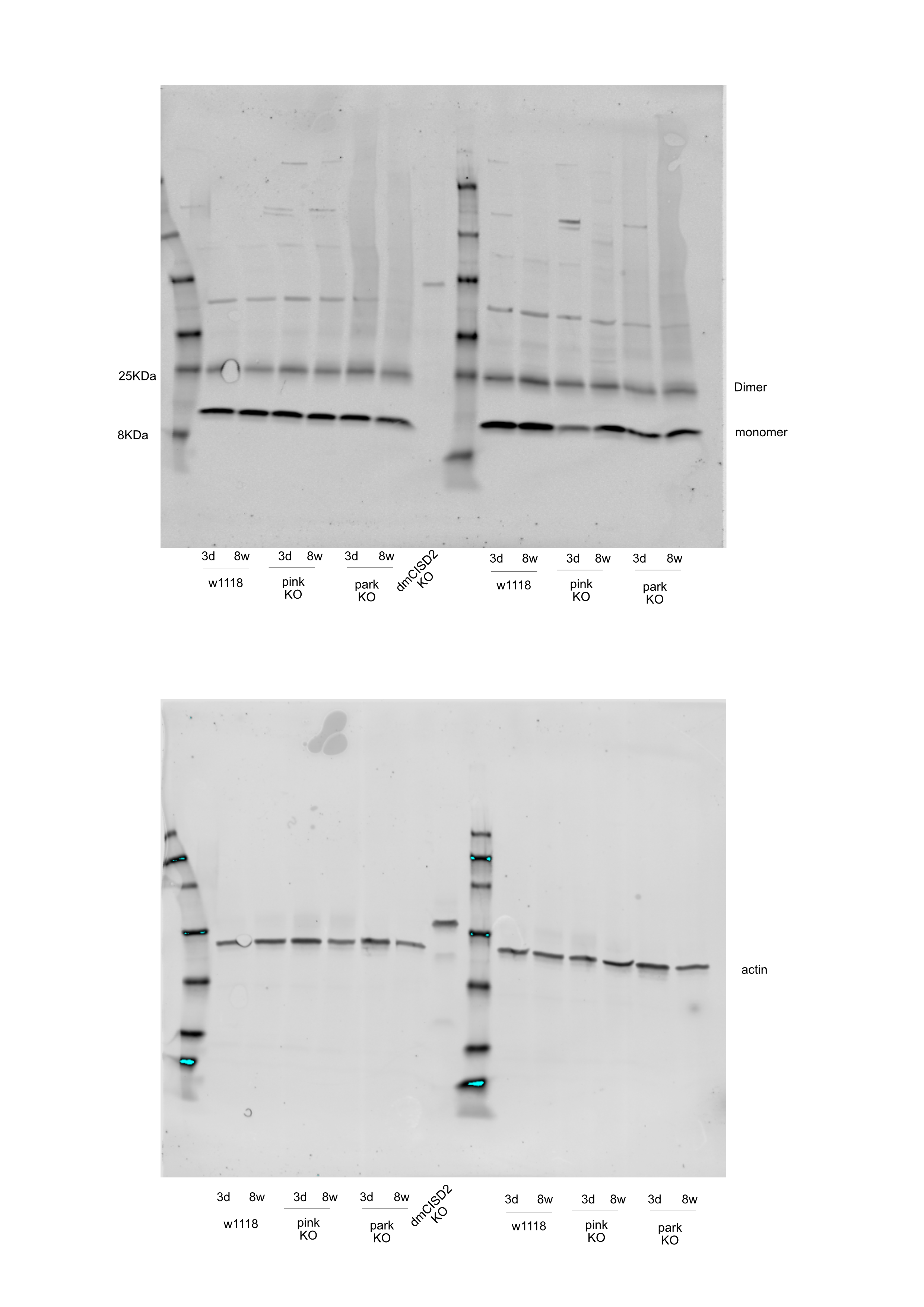

Supplement: Figure 6—source data 1. [file elife-97027-fig6-data1.zip › Figure 6 - source data 1/figure 6 - 6 C┬┤:D┬┤- labeled.png]

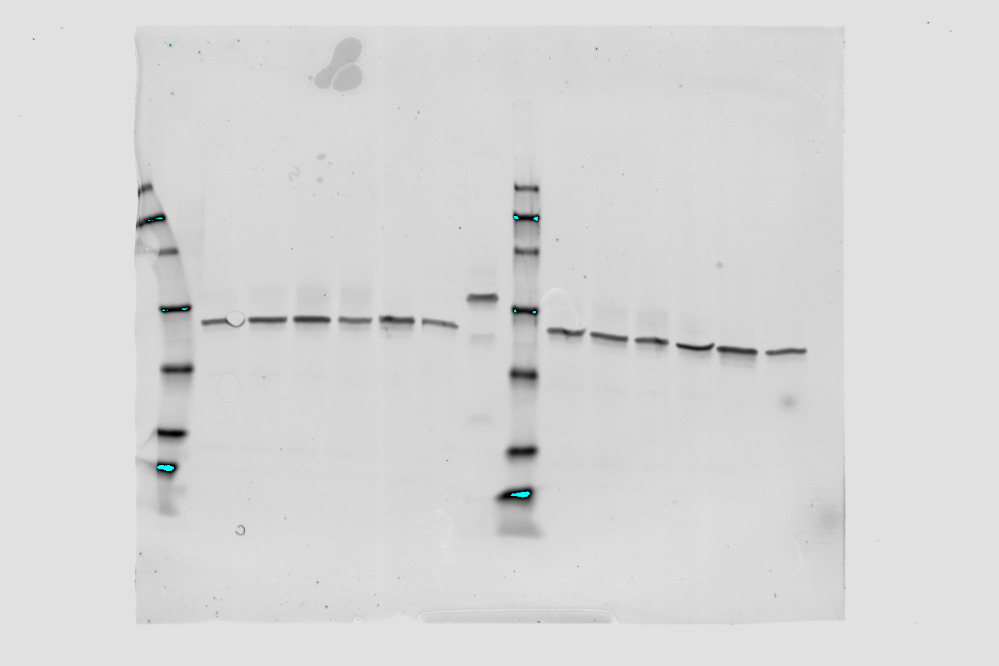

Supplement: Figure 6—source data 1. [file elife-97027-fig6-data1.zip › Figure 6 - source data 1/figure 6 - 6 C┬┤:D┬┤- raw 1.tif]

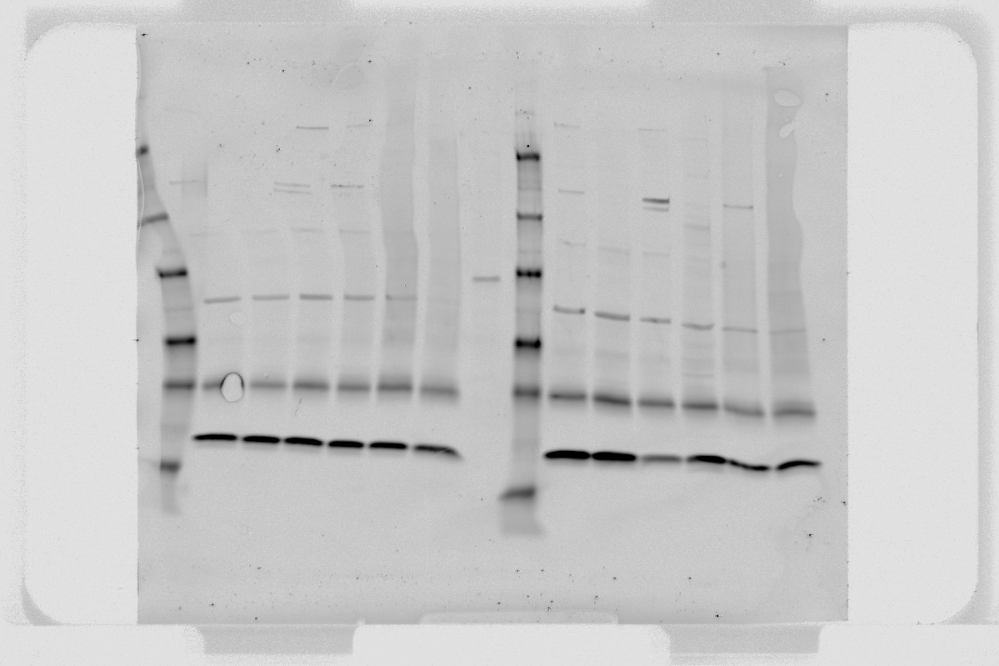

Supplement: Figure 6—source data 1. [file elife-97027-fig6-data1.zip › Figure 6 - source data 1/figure 6 - 6 C┬┤:D┬┤- raw 2.tif]
